# Targeting *ROCK2* to Restore Epileptic Synaptic Networks via Mitophagy Activation: Insights from Translational Imaging of SV2A In Vivo

**DOI:** 10.1002/advs.202508161

**Published:** 2025-08-29

**Authors:** Ling Xiao, Jing Wang, Bei Chen, Jinhui Yang, Fangyu Wu, Chunyao Zhou, Yifei Zhang, Zhiquan Yang, Dingyang Liu, Lei Tian, Jianhua Yu, Fei Han, Yongxiang Tang, Li Feng, Shuo Hu

**Affiliations:** ^1^ Department of Nuclear Medicine Xiangya Hospital Central South University Changsha Hunan 410008 China; ^2^ Department of Neurology Xiangya Hospital Central South University Changsha Hunan 410008 China; ^3^ National Clinical Research Center for Geriatric Diseases Xiangya Hospital Central South University Changsha Hunan 410008 China; ^4^ School of Basic Medical Sciences and Forensic Medicine Hangzhou Medical College Hangzhou Zhejiang 311399 China; ^5^ Department of Neurosurgery Xiangya Hospital Central South University Changsha Hunan 410008 China; ^6^ GE Healthcare Shanghai 210000 China; ^7^ Division of Hematology & Oncology Department of Medicine School of Medicine University of California Irvine Irvine CA 92697 USA; ^8^ Department of Pharmacy College of Biology Hunan University Changsha Hunan 410082 China; ^9^ Key Laboratory of Biological Nanotechnology of National Health Commission Xiangya Hospital Central South University Changsha Hunan 410008 China

**Keywords:** [^18^F]SynVesT‐1, epilepsy, imaging transcriptomics, PET, *ROCK2*, SV2A

## Abstract

Temporal lobe epilepsy (TLE) is increasingly recognized as a network‐level disorder, with contemporary strategies shifting focus from localized epileptic lesions to targeting dysfunctional epileptogenic networks. Leveraging recent advancements in neuroimaging genetics and the growing understanding of brain network remodeling in epilepsy, partial least squares regression is employed to integrate the altered synaptic connectome in TLE patients with a human transcriptomics dataset. The findings reveal a strong association between disruptions in synaptic density similarity networks and the spatial transcriptional profiles of TLE risk genes, identifying Rho‐associated protein kinase 2 (*ROCK2*) as a pivotal gene. In TLE mouse models, treatment with a *ROCK2*‐specific inhibitor mitigates synaptic and neuronal loss, enhances network efficiency within the synaptic density connectome, and significantly reduces seizure frequency. Additionally, transcriptome profiling identifies multiple autophagy‐related pathways, and electron microscopy verifies that the administration of the *ROCK2* inhibitor restores mitochondrial autophagy and reduces the accumulation of damaged mitochondria. These findings suggest that *ROCK2* inhibitors may modulate synaptic networks and mitochondrial dysfunction, offering promising therapeutic potential for the treatment of TLE. This study provides novel insights into the genetic and molecular mechanisms driving epileptic network dysfunction and highlights *ROCK2* as a compelling target for translational epilepsy research.

## Graphical abstract

By integrating synaptic vesicle glycoprotein 2A PET imaging and transcriptomics, this study identifies Rho‐associated protein kinase 2 (*ROCK2*) as a critical regulator of synaptic network dysfunction in temporal lobe epilepsy. Pharmacological *ROCK2* inhibition restores synaptic density and enhances mitophagy, thereby reducing seizures and neuronal damage. These findings support *ROCK2* as a promising therapeutic target for epilepsy network modulation.

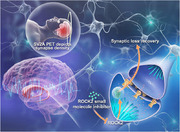

## 1 Introduction

The human brain is a highly intricate network comprising interconnected regions that function in concert to sustain normal physiological operations.^[^ ^1^ ^]^ Epilepsy, a debilitating neurological disorder, disrupts this finely tuned connectivity and manifests as a prototypical network disorder.^[^ ^2^, ^3^, ^4^ ^]^ Recent advancements in connectomics—the study of brain networks through structural and functional imaging—have provided compelling evidence of widespread remodeling in brain networks of individuals with epilepsy, particularly in temporal lobe epilepsy (TLE).^[^ ^5^, ^6^, ^7^ ^]^ TLE is the most common form of focal epilepsy and is frequently characterized by hippocampal sclerosis and the propagation of epileptic activity beyond the temporal lobe. During seizures, hypersynchronous discharges travel through long‐range corticocortical and corticothalamic connections, inducing global neuronal dysfunction.^[^ ^8^, ^9^ ^]^ Even during interictal periods, seizure‐driven pathophysiological changes lead to persistent abnormalities in brain network organization.^[^ ^10^, ^11^ ^]^ Disruptions in network synchronization and the formation of recurrent excitatory circuits are critical to the generation and maintenance of seizures, highlighting the urgent need to understand brain network remodeling mechanisms and their contribution to TLE progression. Such insights could pave the way for novel therapeutic interventions targeting network‐level dysfunction.

Advances in neuroimaging genetics have emerged as a transformative approach to decoding the molecular basis of brain network remodeling.^[^ ^12^ ^]^ Longitudinal imaging genetics studies have linked structural alterations such as accelerated cortical thinning to the expression of genes implicated in dendritic reorganization and synapse loss.^[^ ^13^ ^]^ Similarly, structural magnetic resonance imaging (MRI) and diffusion‐weighted imaging have revealed reduced gray matter volume and impaired white matter tracts in genetically generalized epilepsy, with a particular focus on frontal and temporal lobes.^[^ ^14^, ^15^ ^]^ Simultaneous electroencephalography‐functional MRI has also revealed impairments in thalamocortical circuits among patients with genetic generalized epilepsy.^[^ ^16^ ^]^ Furthermore, several studies have indicated that patients with epilepsy and their asymptomatic relatives exhibit similar patterns of structural and functional brain alterations, suggesting a potential epigenetic substrate driving epileptic network reconfiguration.^[^ ^17^, ^18^ ^]^ Conversely, recurrent seizures can induce transcriptional changes, further remodeling epileptic circuits.^[^ ^3^, ^4^, ^7^, ^19^ ^]^ Therefore, understanding the bidirectional relationship between genetics and network alterations provides an opportunity to unravel the pathophysiological mechanisms underlying epilepsy.

Because synapses serve as the fundamental units for communication between neurons,^[^ ^20^ ^]^ changes in synaptic density and function are tightly coupled with circuit‐level impairments and network remodeling in TLE. Recent advances in molecular imaging techniques such as synaptic vesicle glycoprotein 2A (SV2A) PET imaging provide a noninvasive tool to interrogate synaptic density in vivo and track synaptic changes across the brain.^[^ ^21^, ^22^ ^]^ SV2A, a presynaptic protein that regulates neurotransmitter release,^[^ ^23^ ^]^ is a validated marker of synaptic functionality.^[^ ^24^, ^25^ ^]^ The loss of SV2A has been linked to various neurological disorders, including epilepsy.^[^ ^26^, ^27^, ^28^, ^29^ ^]^ Moreover, SV2A has been identified as a binding site for the antiseizure medication levetiracetam in the brain.^[^ ^30^ ^]^ SV2A PET imaging using [^18^F]SynVesT‐1 can quantify brain synapse density in vivo and track dynamic changes in brain synapses across clinical populations.^[^ ^22^, ^31^, ^32^, ^33^, ^34^ ^]^ Previous studies have utilized SV2A PET imaging to identify synaptic loss in the seizure‐onset zone of patients with TLE.^[^ ^33^, ^34^, ^35^, ^36^ ^]^ While these studies emphasized changes in SV2A‐specific binding in localized brain regions of TLE patients, they did not comprehensively consider interactions between different brain regions and network topologies. Consequently, alterations in synaptic density at the network level remain unclear. The synaptic connectome obtained through PET could extend the previous results of tracking global brain SV2A deficiency, facilitating the mapping of synaptic networks throughout the brain in TLE. Simultaneously, the burgeoning availability of transcriptomic datasets, such as the Allen Human Brain Atlas (AHBA), presents an unprecedented opportunity to link macroscopic imaging to microscopic gene expression.^[^ ^37^, ^38^, ^39^, ^40^ ^]^ These data allow for voxel‐to‐gene spatial mapping, enabling researchers to identify genes whose spatial expression profiles align with imaging features observed in neurological disorders. However, an integrated approach specifically linking brain‐wide synaptic connectomics to regional gene expression patterns has yet to be applied systematically in TLE. Such an integrative framework would illuminate genetic and molecular mechanisms shaping network alterations, offering novel insights into the complex organization of epilepsy networks.

**Scheme 1:**
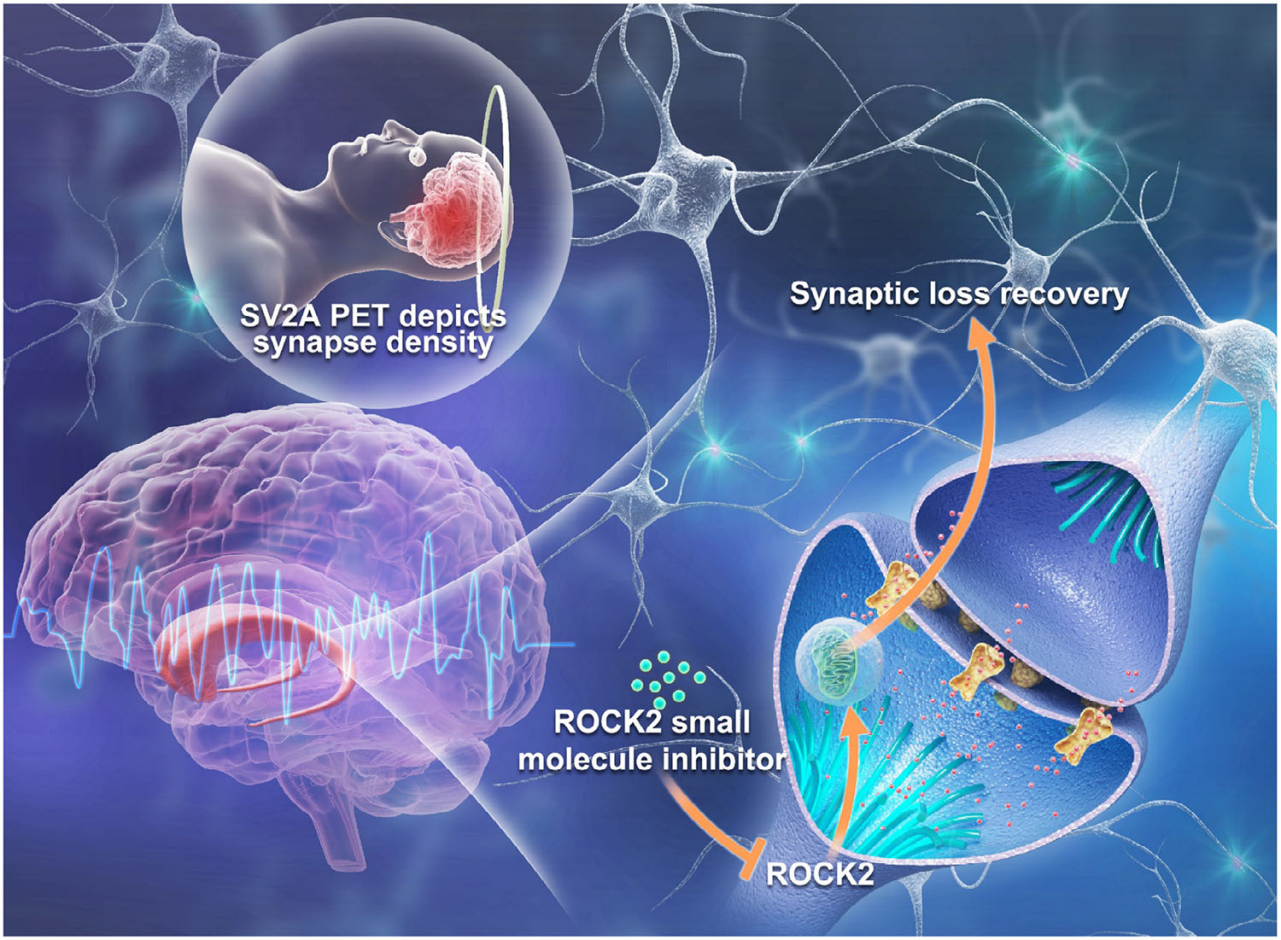
Synaptic vesicle glycoprotein 2A (SV2A) PET visualizes synaptic density changes in temporal lobe epilepsy (TLE). Pharmacological inhibition of Rho‐associated protein kinase 2 (*ROCK2*) mitigates synaptic loss and mitochondrial dysfunction, potentially restoring synaptic integrity and offering a promising therapeutic strategy for TLE.

In this study, we employed partial least squares (PLS) regression to integrate the altered synaptic connectome of TLE patients with a human transcriptomics dataset, identifying Rho‐associated protein kinase 2 (*ROCK2*) as a pivotal gene involved in synaptic network remodeling in individuals with TLE. Subsequently, we established a mouse model of TLE and found *ROCK2* specific overexpression accompanied by synaptic loss in hippocampal and cortical regions. Treatment with belumosudil, a selective small‐molecule *ROCK2* inhibitor, partially reversed synaptic and neuronal loss, enhanced network efficiency within the synaptic density connectome, and reduced seizure frequency. Additionally, transcriptome profiling identified multiple autophagy‐related pathways, and electron microscopy verified that the *ROCK2* inhibitor enhances mitochondrial autophagy and decreases the accumulation of damaged mitochondria. These findings delineate a novel imaging–transcriptomic relationship between *ROCK2* and brain‐wide synaptic changes in TLE, suggesting that *ROCK2*‐specific inhibitors may regulate synaptic networks by enhancing mitochondrial autophagy and promoting synaptic remodeling in TLE, highlighting their potential as a therapeutic strategy for epilepsy (**Scheme** **1**).

## 2 Results

### 2.1 [^18^F]SynVesT‐1 PET Targeting the SV2A Reveals Altered Synaptic Density Similarity Networks in Patients with TLE

This study integrated neuroimaging and transcriptomics data to explore the relationship between alterations in synaptic density similarity networks in TLE and the expression of risk genes. The analyses included one PET cohort and two independent transcriptomic cohorts. A total of 25 TLE patients (8 females, 17 males; 31.16 ± 11.30 years) and 23 healthy controls (9 females, 14 males; 30.48 ± 14.65 years) underwent [^18^F]SynVesT‐1 PET imaging. Clinical characteristics of the PET cohort are provided in Table S1 (Supporting Information). Transcriptome analyses were conducted on hippocampal dentate gyrus tissues from 6 TLE patients and 6 postmortem controls (gender‐matched; 3 females and 3 males in each group; Table S2, Supporting Information). To broaden the analysis of TLE risk genes across cortical regions, transcriptional data from a large published TLE dataset (*n* = 161)^[^ ^41^ ^]^ derived from the temporal neocortex were also incorporated.

**Figure 1:**
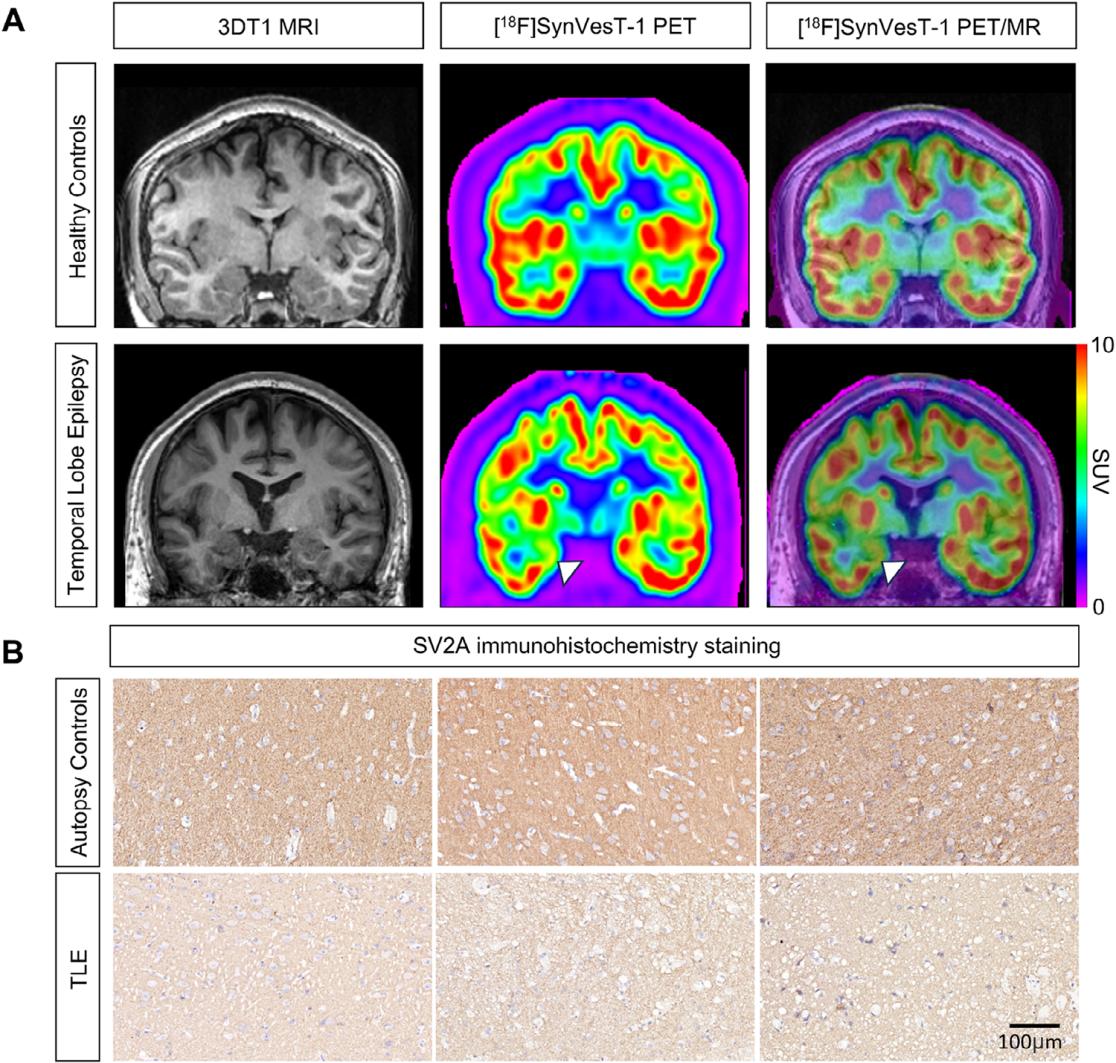
[^18^F]SynVesT‐1 PET/MR and immunohistochemistry staining of patients with TLE. A) Representative coronal T1‐weighted MR (left), [^18^F]SynVesT‐1 PET (middle), and fused [^18^F]SynVesT‐1 PET/MR (right) image in a healthy control (HC) (top) and a patient with TLE (bottom). An evident reduction of synaptic density in the hippocampus of participants with TLE was noted compared with controls (the arrowhead denotes the right hippocampus). B) Immunohistochemistry staining in the ipsilateral hippocampus of patients with TLE showed lower SV2A expression.

**Figure 2:**
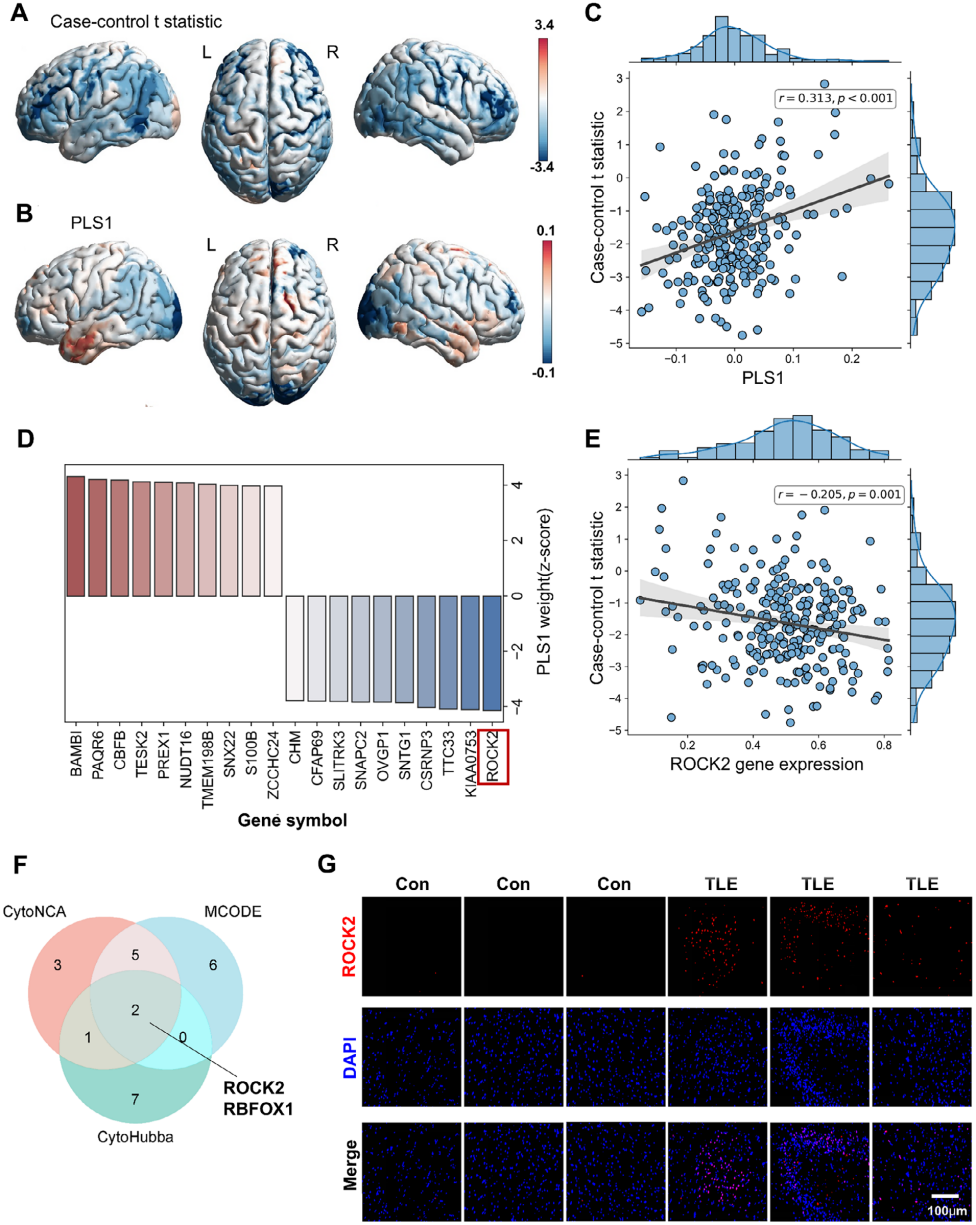
TLE risk gene expression profiles associated with synaptic density network differences. A) TLE–HC nodal connectivity strength difference map and B) weighted TLE risk gene expression map of regional the first PLS component (*PLS1)* scores in the whole brain. C) Scatterplot of regional *PLS1* scores (a weighted sum of 5451 gene expression scores) and case–control differences in nodal connectivity strength of synaptic density network (*r* = 0.313, *p* < 0.001). D) An illustrative example of the weights assigned to representative genes on *PLS1*. Genes that are strongly E) negatively weighted on *PLS1* (e.g., *ROCK2*) correlate negatively with case–control differences of nodal strength in the synaptic density network (*r* = −0.205, *p* = 0.001). F) Venn diagram demonstrates overlapping genes between different calculation methods of CytoNCA, MCODE, and Cytohubba. G) Immunofluorescence staining of *ROCK2* in postmortem control specimens and surgically resected hippocampal tissues obtained from TLE patients.

Representative PET images (**Figure** **1**A) and immunohistochemistry staining (Figure 1B) showed reduced SV2A binding in the ipsilateral temporal lobe of TLE patients, whereas no abnormalities were observed in controls. To examine network topology and brain interactions, a synaptic density connectome (246 × 246 regions) was generated by calculating Kullback–Leibler divergence similarity (KLS)^[^ ^42^ ^]^ of the probability density functions of standardized uptake value ratio (SUVR) across brain regions in [^18^F]SynVesT‐1 PET images. Differences in nodal connectivity strength, a key graph‐theoretical metric for complex networks, were analyzed between cases and controls (**Figure** **2**A). Compared to controls, TLE patients exhibited reduced nodal connectivity strength, indicating impaired connectivity within the synaptic density similarity networks. Specifically, reductions in synaptic density similarity networks were predominantly observed in the temporal lobe, amygdala, hippocampus, basal ganglia, precuneus, and frontal gyrus (*p* < 0.01, FDR corrected; Table S3, Supporting Information).

### 2.2 The Changes in Synaptic Density Similarity Networks in Patients with TLE Are Linked to Risk Gene Expression, with *ROCK2* Identified as a Pivotal Gene

To identify differentially expressed risk genes related to TLE, we obtained brain gene expression data from hippocampal dentate gyrus tissues and a previously published temporal cortical transcriptomic dataset by Guelfi et al.^[^ ^41^ ^]^ To investigate the spatial relevance of TLE‐related risk genes in network alterations, we analyzed the correlation between case–control differences in nodal connectivity strength and TLE‐related gene expression maps. Utilizing the AHBA, a comprehensive whole‐brain transcriptomic reference, we mapped the significantly dysregulated genes to derive spatial expression profiles of TLE risk genes. Then, we used PLS regression to reveal that gene expression in brain regions exhibited significant correlations with nodal connectivity strength differences in the synaptic density similarity networks (*r* = 0.313, *p* < 0.001), with the first PLS component (*PLS1*) scores from the temporal lobe region displaying the largest weight (Figure 2B,C). By conducting 5000 permutation tests on the PLS regression model and applying *z*‐score transformations of the regression model weights, we identified 237 genes with normalized *PLS1* weights of *z* > 3 (*PLS+*) and 100 genes with normalized *PLS1* weights of *z* < −3 (*PLS−*), collectively referred to as the *PLS1* gene set. Among these, the *BAMBI* gene exhibited the highest positive *PLS1* weight (*r* = 0.217, *p* < 0.001), and *ROCK2* displayed the highest negative *PLS1* weight (*r* = −0.205, *p* < 0.001) (Figure 2D,E). *PLS1* genes with positive weights were associated with increased nodal connectivity strength in the synaptic density similarity networks among TLE patients compared to controls. By contrast, those with negative weights were linked to decreased nodal connectivity strength.

Next, functional annotations and interaction analyses were performed for the *PLS1* gene set to explore pathway enrichments and genetic interactions associated with network topological changes. We used Metascape to align the Gene Ontology (GO) biological processes with the *PLS−* gene set. After correcting for enrichment terms (*p* < 0.01) and excluding discontinuous enrichment clusters, the top 30 significant enrichment terms were illustrated in Figure S1A (Supporting Information) (*p* < 0.05, FDR corrected). The GO enrichment analysis indicated that the *PLS−* genes were particularly enriched for biological processes involved in synaptic transmission (e.g., modulation of chemical synaptic signaling, regulation of transsynaptic signaling), and synapse‐related cellular components (e.g., postsynaptic density, asymmetric synapse, postsynaptic specialization, neuron to neuron synapse, postsynapse, glutamatergic synapse), as well as molecular functions of kinase activity (e.g., protein serine/threonine kinase activity, protein serine kinase activity, GTPase activator activity, GTPase binding, and GTPase activator activity) (Figure S1A, Supporting Information). DisGeNET analysis further highlighted “Epilepsies, Partial” as the most enriched term (with a *p* < 0.01, a minimum count of 3, and an enrichment factor > 1.5) (Figure S1B, Supporting Information). We also assessed the *PLS+* gene set for significant GO enrichment, which was related to biological processes involving lipid biosynthesis and metabolism (Figure S2A, Supporting Information) and associated “Amyloidsis” as the most significant DisGeNET term (Figure S2B, Supporting Information). These results suggest that the *PLS−* gene set may have specific associations with epilepsy and synaptic transmission.

To identify the pivotal gene, we utilized GeneMANIA (http://genemania.org) to construct gene–gene interaction networks. The interactive relationships among key genes within the entire network were determined using Cytoscape plugins, specifically MCODE, CytoNCA, and CytoHubba. By employing the MCODE algorithm with default parameters (node score cutoff = 0.2, degree cutoff = 2, *k*‐core = 2, maximum depth set to 100), we identified five functional modules: Module 1 (MCODE score = 10.167), Module 2 (MCODE score = 4.200), Module 3 (MCODE score = 3.600), Module 4 (MCODE score = 3.000), and Module 5 (MCODE score = 2.800). Functional Module 1, which exhibited the highest MCODE score, contained 13 genes. To further prioritize hub genes, we employed CytoHubba, extracting the top 10 genes through four computational algorithms (EPC, MCC, MNC, and Stress). Additionally, the application of CytoNCA algorithms (Degree, Eigenvector, Betweenness, and Closeness) identified 11 key genes. A Venn diagram of hub genes shared across multiple analyses identified *ROCK2* and *RBFOX1* as significant central nodes within the network (Figure 2F). Notably, *ROCK2* demonstrated the highest negative *PLS1* weight and was consistently prioritized as a key hub gene, establishing it as a pivotal target for further investigation.

To investigate *ROCK2* expression patterns, we performed immunofluorescence staining on both postmortem control specimens and surgically resected hippocampal tissues from TLE patients (Figure 2G). Quantitative analysis revealed a significant increase in *ROCK2*‐positive area in the brain tissue of patients with TLE compared to postmortem controls (*p* < 0.0001; Figure S3, Supporting Information).

### 2.3 *ROCK2* Inhibition Ameliorates Neuronal and Synaptic Loss, Reduces Episode Intensity, and Improves Survival in Acute TLE Mouse Models

**Figure 3:**
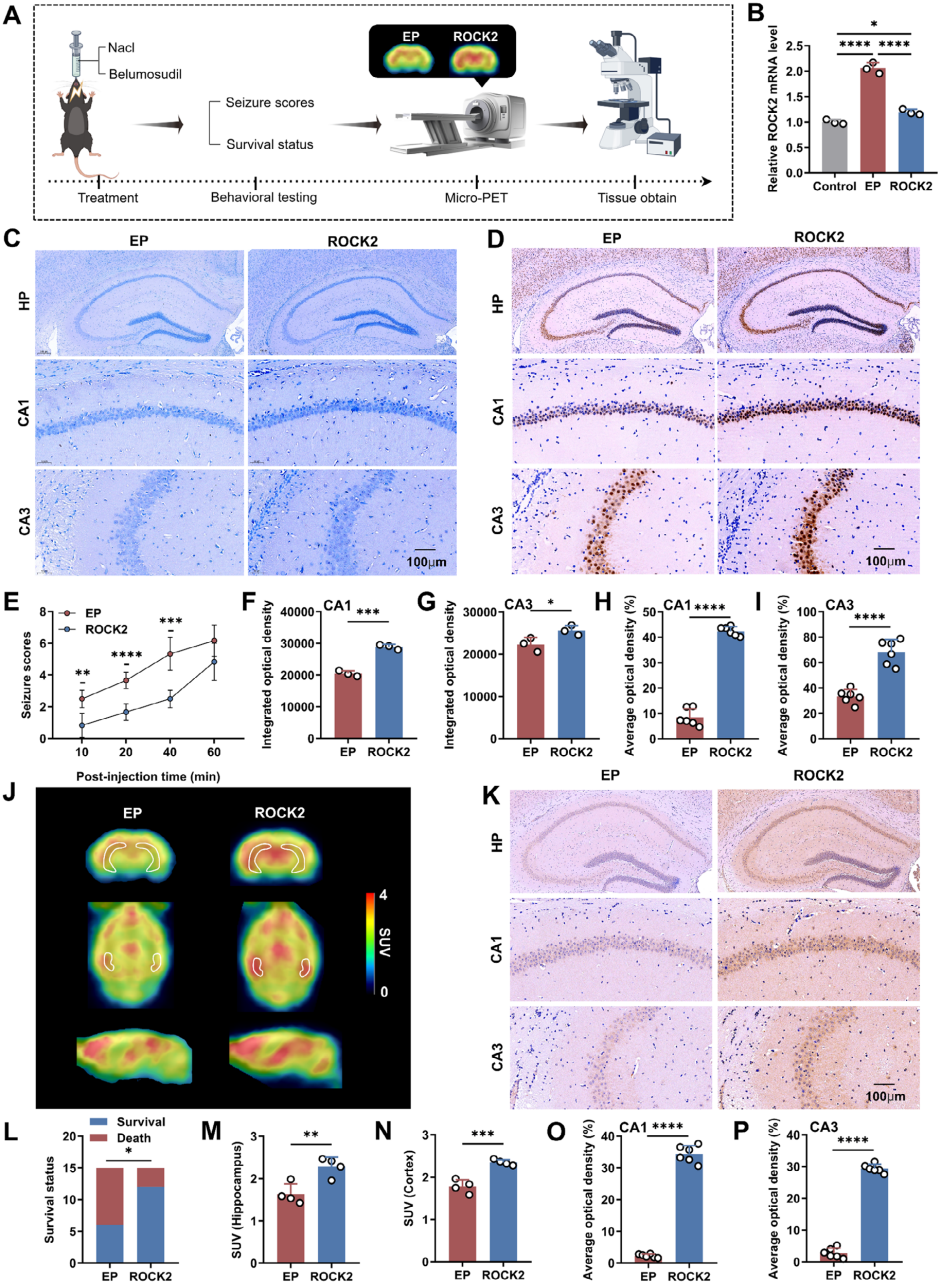
Effects of *ROCK2* inhibitor on TLE mice in the acute phase. A) Flow chart of the experimental scheme. B) The expression levels of *ROCK2* in the hippocampal tissues of mice in the control group, EP group, and *ROCK2* group. C,F,G) Representative images and quantification analysis of Nissl staining in the hippocampal CA1 and CA3 regions of the EP and *ROCK2* groups. D,H,I) Representative images and quantification analysis of NeuN staining in the hippocampal CA1 and CA3 regions of the two groups. E) Change in mean seizure intensity over time in the two groups. J,M,N) Representative coronal, axial, and sagittal SUV images of [^18^F]SynVesT‐1 PET (from 30 to 60 min) in the two groups, and SUV values in the hippocampus and cortex regions. The structures outlined in white are the bilateral hippocampus. K,O,P) Representative images and quantification analysis of SV2A expression in hippocampal subregions, including CA1 and CA3, in the two groups. L) Survival status of mice 2 h after injection of pilocarpine. Error bars represent standard deviation (SD). *p < 0.05, **p < 0.01, ***p < 0.001, ****p < 0.0001.

To validate *ROCK2* as a therapeutic target for TLE, we established pilocarpine‐induced acute TLE mouse models and randomly assigned mice into two groups: one group received the *ROCK2* inhibitor belumosudil (the *ROCK2* group), and the other received saline as a control (the epilepsy model (EP) group). Quantitative real‐time polymerase chain reaction (PCR) analysis showed that *ROCK2* mRNA expression was significantly upregulated in EP mice compared to controls, but markedly reduced following treatment with *ROCK2* inhibitor belumosudil (**Figure** **3**B). To evaluate the therapeutic effects of *ROCK2* inhibitors, we performed behavioral scoring, in vivo micro‐PET imaging, and histological analysis (Figure 3A).

To assess seizure intensity, mice were monitored and scored at 10, 20, 40, and 60 min postpilocarpine injection according to the modified racine scale.^[^ ^43^ ^]^ Line graphs were generated from the tabulated data. A repeated‐measures ANOVA revealed an interaction between *ROCK2* inhibitor treatment and time, impacting seizure intensity. Over time, the seizure intensity score exhibited a gradual increase. The *ROCK2* group demonstrated lower scores compared to the EP group at equivalent time points, indicating that *ROCK2* inhibitor could delay the escalation of seizure intensity (Figure 3E). Analysis of the survival status of mice at the final score, after accounting for the sample size, revealed significantly reduced mortality following *ROCK2* inhibitor treatment, suggesting that belumosudil improved survival rates in mice with acute epilepsy (Figure 3L). Thus, the *ROCK2* inhibitor reduced episode intensity, enhanced prognosis, and prolonged survival in the acute TLE mouse model.

To examine the neuroprotective effects of *ROCK2* inhibitors against epilepsy, a morphological analysis of the hippocampal organotypic tissue of mice was performed using Nissl and NeuN staining. Both staining techniques demonstrated a significant increase in the number of preserved neurons in the CA1 and CA3 regions of the hippocampus of the *ROCK2* group following seizure activity, relative to the EP group (Figure 3C,D,F–I).

To investigate the impact of *ROCK2* inhibitors on synaptic density in mouse brains, we first performed in vivo SV2A PET imaging. SV2A is ubiquitously expressed in cerebral gray matter, making it challenging to identify a suitable reference region within the mouse brain.^[^ ^44^ ^]^ Consequently, we compared standardized uptake value (SUV) rather than SUVR across mouse brain regions. The results showed significantly elevated SUV in the hippocampus and cortex of the *ROCK2* group compared to the EP group (Figure 3J,M,N). Subsequent SV2A immunohistochemical staining further corroborated these findings, displaying an increased SV2A expression in the hippocampal CA1 and CA3 regions after treatment with *ROCK2* inhibitor (Figure 3K,O,P). Collectively, these results indicate that *ROCK2* inhibitor treatment in the acute phase can ameliorate neuronal and synaptic loss, reduce episode intensity, and increase survival rates.

### 2.4 *ROCK2* Inhibition Ameliorates Neuronal and Synaptic Loss, Reduces Seizure Frequency, and Improves Cognitive Function in Chronic and Drug‐Resistant TLE Mouse Models

**Figure 4:**
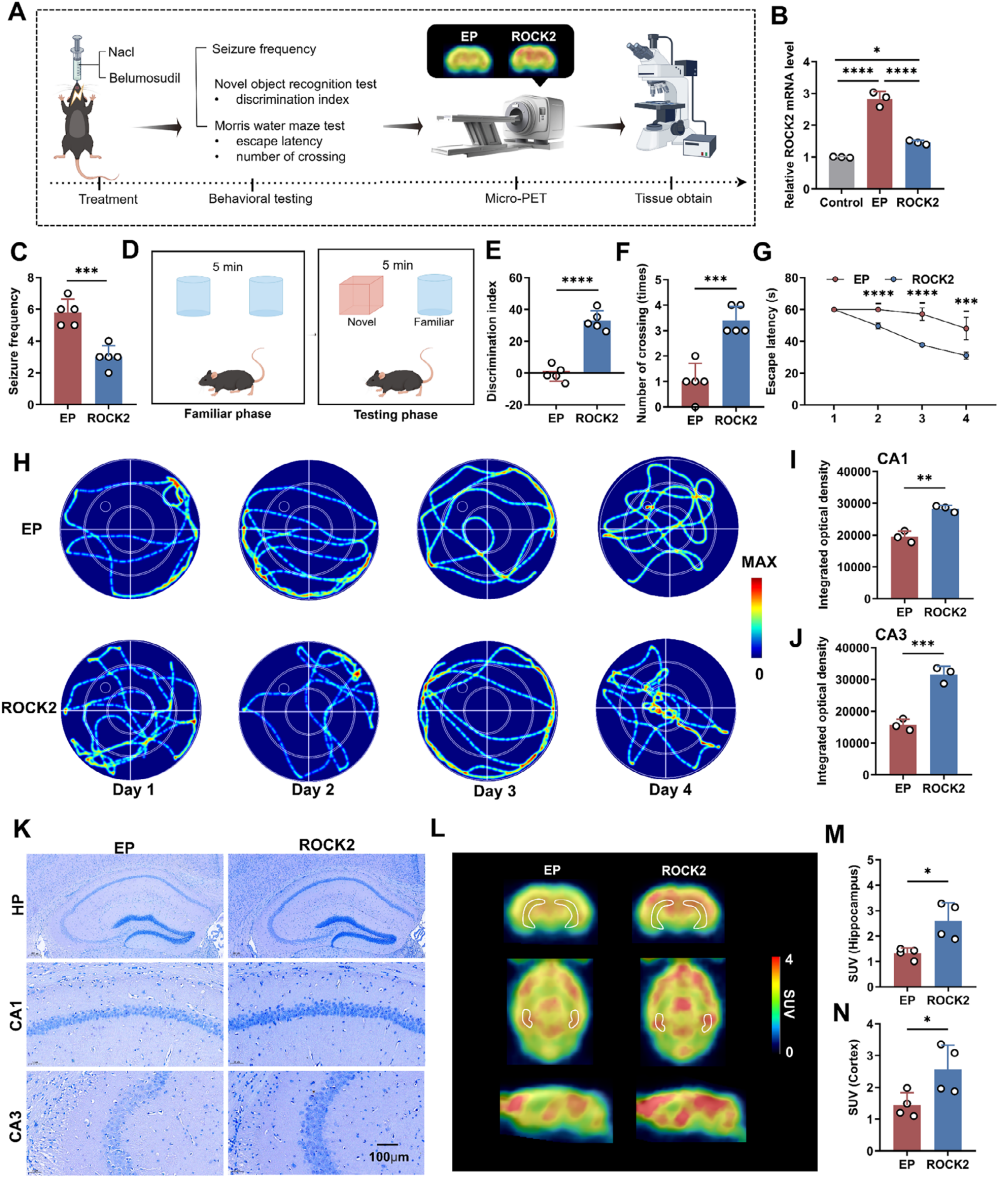
Effects of *ROCK2* inhibitor on TLE mice in the chronic phase. A) Flow chart of the experimental scheme. B) The expression levels of *ROCK2* in the hippocampal tissues of mice in the control group, EP group, and *ROCK2* group. C) The average number of seizures per day in mice. D) Novel object recognition (NOR) test. In the testing phase, the cylinder is a familiar object, whereas the cube is a novel object. E) The discrimination indices for NOR tests of mice with TLE or those treated with *ROCK2* inhibitor. F) Number of times mice crossed the target location during the probe test. G) Escape latency of mice to find the emerged platform during the training trial. H) Indication of swimming traces from days 1 to 4 in the two groups. The small circle in quadrant II represents the platform, and the large circle represents the pool edge. I–K) Representative images and quantification analysis of Nissl staining in the hippocampal CA1 and CA3 regions of the EP and *ROCK2* groups. L–N) Representative coronal, axial, and sagittal SUV images of [^18^F]SynVesT‐1 PET (from 30 to 60 min) in the two groups, and SUV values in the hippocampus and cortex regions. The structures outlined in white are the bilateral hippocampus. Error bars represent SD. *p < 0.05, **p < 0.01, ***p < 0.001, ****p < 0.0001.

To replicate key features of human TLE, we investigated the therapeutic effects of *ROCK2* inhibitors in chronic and drug‐resistant TLE mouse models. Behavioral evaluations, cognitive assessment, in vivo micro‐PET imaging, and histological analyses were performed to determine treatment efficacy (**Figure** **4**A). Quantitative real‐time PCR revealed that *ROCK2* mRNA expression was significantly upregulated in the EP group compared to the control mice, but substantially reduced following *ROCK2* inhibitors (Figure 4B).

To assess the impact of *ROCK2* inhibitors on seizure burden, mice were monitored via continuous video recording for one week during the chronic stage. Seizure frequency was significantly reduced in the *ROCK2*‐treated group compared to the EP group (Figure 4C), suggesting effective seizure control and long‐term therapeutic potential of *ROCK2* inhibitors.

To examine cognitive improvements, we assessed nonspatial recognition memory and spatial learning and memory using the novel object recognition test and the Morris water maze, respectively. In the novel object recognition test, *ROCK2* inhibitors significantly improved the ability of TLE mice to recognize new and familiar objects compared with the untreated mice (Figure 4D,E). Similarly, in the Morris water maze, *ROCK2* inhibition resulted in shorter escape latencies and increased crossings through the target site, indicating superior memory retention and spatial learning (Figure 4F,G). And representative swimming traces concerning 4 days of training trials among different groups are shown (Figure 4H). These results underscore the positive effects of *ROCK2* inhibition on cognitive deficits associated with TLE.

Histological analyses of hippocampal tissue using Nissl and NeuN staining demonstrated that *ROCK2* inhibitor treatment markedly increased the number of surviving neurons in the CA1 and CA3 hippocampal regions, consistent with findings from acute TLE models (Figure 4I–K and Figure S4A,C,D (Supporting Information)). These results further confirm the neuroprotective role of *ROCK2* inhibition in mitigating seizure‐induced neuronal loss over chronic disease progression.

To evaluate synaptic density, in vivo SV2A PET imaging and SV2A immunohistochemical staining were performed. PET imaging revealed significantly elevated SUVs in the hippocampus and cortex of *ROCK2*‐treated mice compared with the EP group (Figure 4L–N). Immunohistochemical analysis confirmed upregulated SV2A expression in the CA1 and CA3 hippocampal regions in the *ROCK2* group (Figure S4B,E,F, Supporting Information), further demonstrating the ability of *ROCK2* inhibitors to restore synaptic integrity.

Collectively, these findings reveal that *ROCK2* inhibitor treatment during the chronic phase effectively reduces seizure frequency, prevents neuronal and synaptic loss, and enhances cognitive function. Consistent results were observed in drug‐resistant TLE models (Figure S5, Supporting Information), supporting the broad therapeutic potential of *ROCK2* inhibitors for chronic and refractory TLE.

### 2.5 *ROCK2* Inhibition Enhances Network Efficiency within the Synaptic Density Connectome

To investigate the effects of *ROCK2* inhibitors on synaptic density connectome efficiency, mean PET values were extracted from all regions of interest (ROIs) in the Ma–Benveniste–Mirrione atlas,^[^ ^45^ ^]^ as previously described.^[^ ^46^ ^]^ Region‐by‐region adjacency matrixes (22 × 22 regions) were then constructed for the EP and *ROCK2* groups. Brain regions were categorized into subcortical networks, including the hippocampus, entorhinal cortex, piriform cortex, hypothalamus, thalamus, amygdala, and olfactory bulb, and cortical networks, comprising the visual cortex, auditory cortex, somatic motor cortex, and somatosensory cortex.

**Figure 5:**
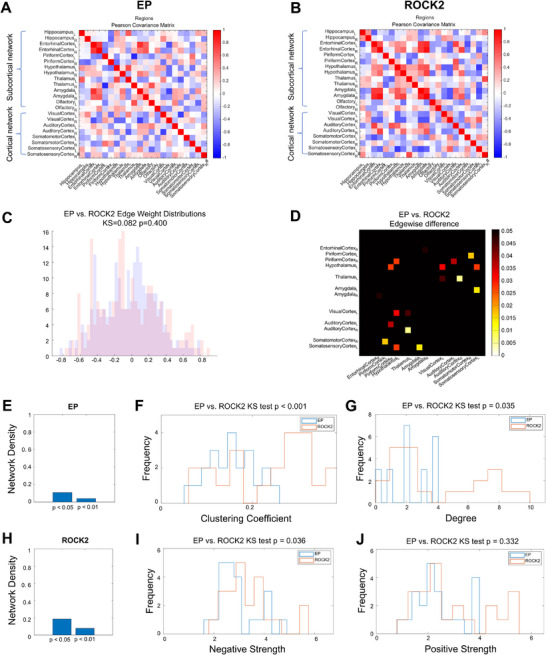
Effects of *ROCK2* inhibitor on network efficiency within the synaptic density connectome. The synaptic density covariance matrices A) EP and B) *ROCK2* groups. Covariance was computed as Pearson correlation of *z*‐scored regional standardized uptake value ratio values across animals within each group. C,D) Covariance network comparisons across genotype. Histograms are distributions of network edge weights statistically compared with a Kolmogorov–Smirnov test at the *p* < 0.05 threshold. Individual edges were tested for group differences with permutation testing (10 000 permutations), with matrices showing uncorrected significance at *p* < 0.05. E–J) Properties of thresholded covariance networks for EP and *ROCK2* groups. (E, H) Network densities of *p* < 0.05 and < 0.01 thresholded respectively. (F) Clustering coefficient, (G) degree, (I) negative, and (J) positive strength nodal distributions of *p* < 0.05 thresholded covariance networks.

The synaptic density covariance matrices are illustrated in **Figure** **5**A,B. Compared to the EP group, the *ROCK2* group exhibited a more structured configuration and organized covariance matrix, indicating improved network organization. Thresholded covariance networks for *p*‐values less than 0.05 and 0.01 are shown in Figure S6 (Supporting Information). No significant differences in the overall distribution of edge weights were observed between the two groups (Figure 5C, Kolmogorov–Smirnov statistic 0.08, *p* > 0.05). However, permutation testing revealed significant edge‐wise differences, particularly in cortical–subcortical connections, including the left thalamus and right auditory cortex, as well as the left amygdala and left somatosensory cortex (Figure 5D). Comparative analysis revealed consistently higher network density values in the *ROCK2* group versus EP group across both edge‐retention thresholds (*p* < 0.05 and *p* < 0.01; Figure 5E,H). The reported *p*‐values reflect threshold‐specific statistical assessments, while the bar graphs present absolute density measurements for each group. Similarly, analysis of node properties revealed that the *ROCK2* group had a significantly higher clustering coefficient (Figure 5F), node degree (i.e., number of connections; Figure 5G), and negative nodal strength compared to the EP group (Figure 5I) (*p* < 0.05, FDR corrected). By contrast, no significant changes were observed in positive nodal strength (Figure 5J). These findings indicate that *ROCK2* inhibition improves both global and local network efficiency within the synaptic density connectome. Additional network properties under a more restrictive threshold (*p* < 0.01) are presented in Figure S7 (Supporting Information).

### 2.6 *ROCK2* Inhibitors Reduce Damaged Mitochondrial Accumulation through the Enhancement of Mitophagy in TLE Mice

**Figure 6:**
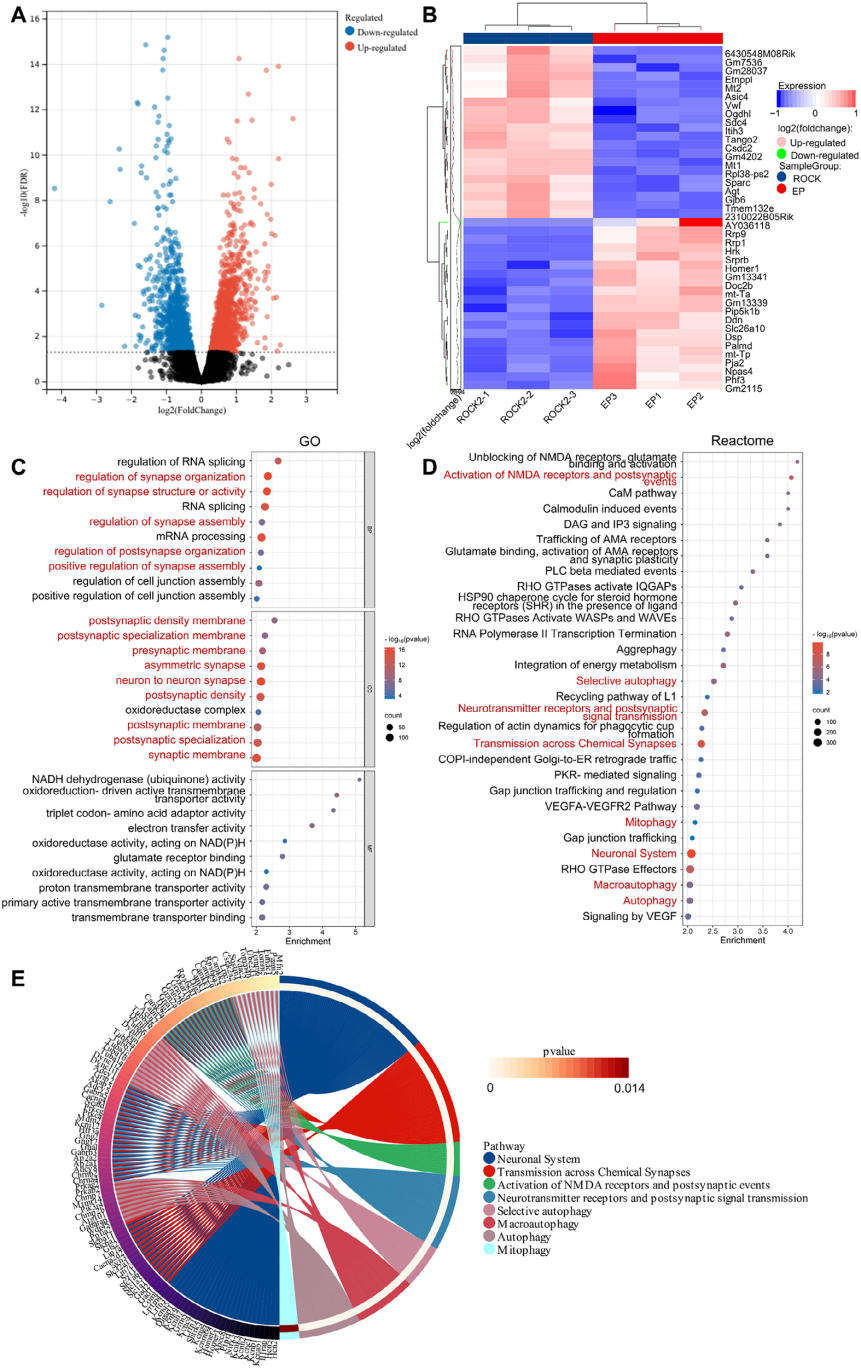
RNA‐seq analysis of hippocampus samples from the TLE mice. A) Volcano plot and B) cluster heatmap of differentially expressed genes. C,D) Bar chart of Gene Ontology (GO) enrichment and Reactome pathway analysis for differentially expressed genes. E) Chord plots illustrate the correspondence between genes and biological processes among the important entries (in red font) in the Reactome pathway analysis.

To further elucidate the mechanisms by which *ROCK2* inhibitors mitigate synaptic loss, hippocampal tissues from both the *ROCK2* and EP groups were collected for mRNA sequencing. Differentially expressed genes (DEGs) were identified based on a fold change > 2 and *p*‐value < 0.05. Compared to the EP group, 1461 genes were upregulated and 1392 genes were downregulated in the *ROCK2* group. The volcano plot (**Figure** **6**A) illustrates the overall DEG distribution, with highly expressed genes in red and lowly expressed genes in blue. The cluster heatmap (Figure 6B) highlights expression patterns, GO enrichment analysis (Figure 6C) revealed significant enrichment of biological processes and cellular components associated with synapses, while Reactome pathway analysis (Figure 6D) identified pathways related to synapses, autophagy, and mitophagy. A detailed visualization of the relationship between altered genes and the corresponding biological processes is presented in Figure 6E (*p* < 0.05, FDR correction). Collectively, these findings implicate *ROCK2* inhibitors in reducing synaptic loss through the regulation of mitophagy.

**Figure 7:**
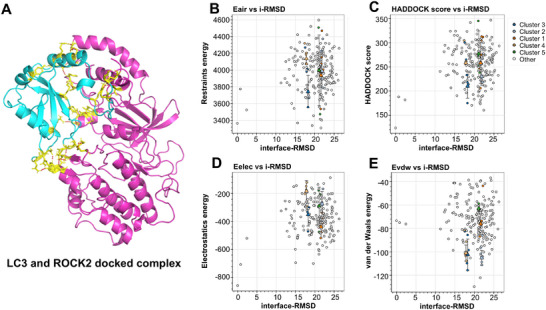
HADDOCK cluster analysis and transmission electron microscopy (TEM) observations of kainic acid (KA) cells and hippocampus samples from the TLE mice. A) Selected *LC3*─*ROCK2* docked complex where *LC3* and *ROCK2* are shown in blue and pink, respectively. The HADDOCK docked models were plotted against their interface‐RMSDs (i‐RMSDs); the color filled triangle corresponds to the individual cluster. B) i‐RMSDs versus air energy (*E*air) plot for CPB─P2×7 complex model. The i‐RMSDs were calculated on the backbone (CA, C, N, O, P) atoms of all residues involved in intermolecular contact using a 10 Å cutoff. C) The HADDOCK scores of clusters were plotted against their i‐RMSDs. The HADDOCK score corresponds to the weighted sum of D) intermolecular electrostatic, E) van der Waals contacts, Desolvation, *E*air, and a buried surface area. HADDOCK differs for the various docking stages (rigid body (it0), semiflexible refinement (it1), and explicit solvent refinement (water)). It uses the following weights: HADDOCKscore‐it0 = 0.01*E*vdw + 1.0*E*elec + 1.0*E*desol + 0.01*E*air – 0.01BSA; HADDOCKscore‐it1 = 1.0*E*vdw + 1.0*E*elec + 1.0*E*desol + 0.1*E*air – 0.01BSA; HADDOCKscore‐water = 1.0*E*vdw + 0.2*E*elec + 1.0*E*desol + 0.1*E*air. And *z*_score_cluster_*i* = (HADDOCKscore‐water – *µ*_all)/*σ*_all.

To explore the relationship between *ROCK2* and mitophagy, we employed automatic high‐ambiguity‐driven biomolecular docking (HADDOCK) to model the interaction of *ROCK2* with *LC3*, a critical autophagosome‐membrane‐associated protein. The results showed that *ROCK2* binds to *LC3* via its *LC3* interaction domain, thereby inhibiting mitophagy (**Figure** **7**A). Belumosudil, a *ROCK2* inhibitor, promotes mitophagy by competitively binding and inactivating *ROCK2*. Among the five docking clusters, Cluster 3, with a HADDOCK score of 209.9 ± 21.3 kcal mol^−1^, a cluster size of 6, an electrostatic energy of −350.0 ± 70.4 kcal mol^−1^, and a *z*‐score of −1.5, was selected as the optimal *LC3*─*ROCK2* complex for subsequent analysis (Figure 7B–E).

**Figure 8:**
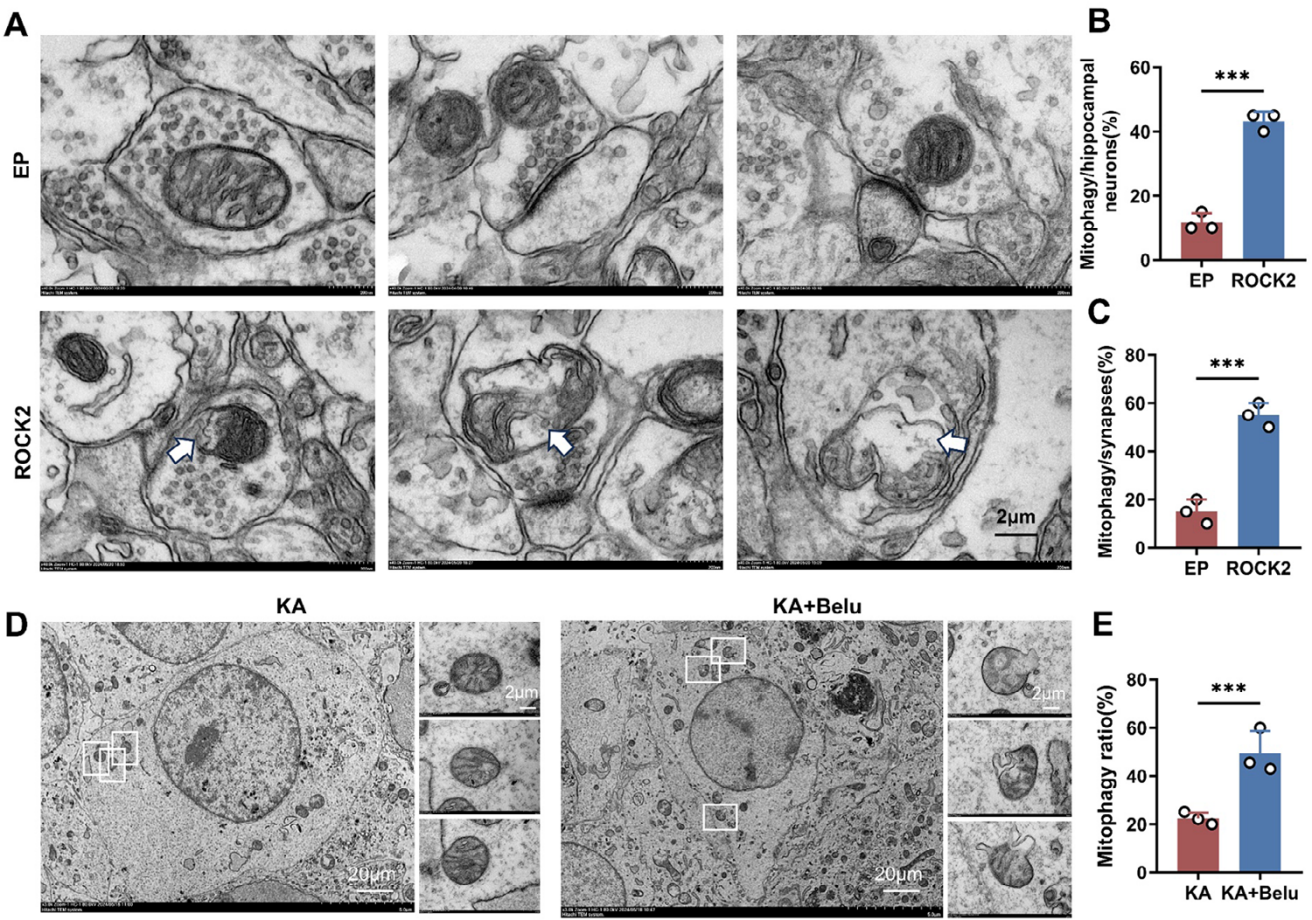
TEM observations of hippocampus samples from the TLE mice and KA neuronal cells. A–C) Representative images and quantification analysis of mitophagy in mice hippocampus by TEM. D,E) Representative images and quantification analysis of mitophagy in KA cells. Error bars represent SD.

To validate these findings, a cellular seizure model was established by inducing kainic acid (KA) in cultural neuronal cells, followed by mitophagy investigation. Transmission electron microscopy (TEM) was used to further investigate mitochondrial integrity in vivo and in vitro. In EP mice, TEM revealed suppressed mitophagy, with significantly reduced proportions of autophagic mitophagy in hippocampal neurons and synapses (**Figure** **8**A–C). By contrast, *ROCK2* inhibition by belumosudil restored mitophagy levels, resulting in increased ratios of mitophagic mitochondria both in hippocampal neurons and synapses. Similar effects were observed in the KA model, where *ROCK2* inhibition reversed KA‐induced suppression of mitophagy, suggesting mitochondrial normalization (Figure 8D,E).

To assess mitochondrial membrane potential, a JC‐1 dye assay was performed. Normal mitochondria aggregation of JC‐1 dye, results in red fluorescence, while disrupted membrane potential shifts fluorescence toward green monomeric signals. Figure S8A (Supporting Information) illustrates that in our coculture model, KA‐induced cells exhibited significantly elevated levels of JC‐1 monomers in neurons, which was mitigated by the administration of the *ROCK2* inhibitor post‐KA activation. Apoptosis or necrosis can disrupt the cell membrane structure. The amount of lactate dehydrogenase (LDH) released directly reflected the integrity of the cell membrane. LDH cytotoxicity results indicated a significant decrease in LDH release in the *ROCK2* group compared to the EP group (Figure S8B, Supporting Information).

### 2.7 Blood–Brain Barrier Permeability and Biosafety Profile of the *ROCK2* Inhibitor

To assess the ability of the *ROCK2* inhibitor to cross the blood–brain barrier (BBB), we employed ACD/Percepta software (version 14.53.0; ACD Labs, Ontario, Canada) to predict its BBB permeability based on chemical structure (Figure S9A, Supporting Information). The predicted transport parameters were as follows: logPS = −1.6, logBB = −0.87, and log(PS × fu, brain) = −3.1. A corresponding scatter plot illustrates the relative position of the compound (green dot) compared to known central‐nervous‐system (CNS)‐active (blue dots) and CNS‐inactive (red dots) drugs, suggesting that the *ROCK2* inhibitor may possess moderate BBB permeability (Figure S9B, Supporting Information).

Finally, histopathological evaluation (Figure S10A, Supporting Information) and serum biomarker analysis (alanine aminotransferase (ALT), aspartate aminotransferase (AST), uric acid (UA), creatinine (CRE), urea (UREA); Figure S10B (Supporting Information)) revealed intact organ morphology and normal hepatic/renal function in *ROCK2*‐inhibitor‐treated TLE mice compared to epileptic controls, demonstrating its favorable biosafety profile at therapeutic doses.

## 3 Discussion

The goal of antiepileptic treatment is to address both the pathogenic factors underlying epilepsy and the associated epileptogenic networks. In this study, we demonstrated that alterations in synaptic density similarity networks in patients with TLE are correlated with the expression of risk genes, among which *ROCK2* emerged as a pivotal regulator. Using acute, chronic, and drug‐resistant TLE mouse models, we found that a *ROCK2*‐specific inhibitor effectively restored synaptic density, enhanced network efficiency within the synaptic density connectome, reduced seizure frequency, and enhanced cognitive performance. Furthermore, transcriptomic profiling revealed significant enrichment of autophagy‐related pathways, while electron microscopy confirmed that *ROCK2* inhibition enhanced mitochondrial autophagy (mitophagy) and reduced the accumulation of damaged mitochondria. Accordingly, we propose that the neuroprotective effects of *ROCK2* inhibition in epilepsy are mediated, at least in part, through the enhancement of neuronal mitophagy, providing a mechanistic basis for its role in modulating both synaptic networks and the progression of epilepsy.

Previous studies have reported a decrease in SV2A expression in the brains of epileptic mice^[^ ^47^, ^48^ ^]^ and resected human brain tissue obtained during epilepsy surgery.^[^ ^49^, ^50^ ^]^ Limited in vivo studies utilizing PET imaging to quantify synaptic density have similarly reported decreased SV2A binding in the ipsilateral hippocampus and mesial temporal lobe of TLE patients.^[^ ^35^, ^36^, ^51^ ^]^ However, these studies primarily focused on localized synaptic changes, without considering potential alterations in global network topology or interactions among spatially distributed brain regions. In the present study, we extended these findings by constructing individual whole‐brain synaptic connectomes based on the SV2A PET images and applying graph theory analysis. Our results revealed that synaptic connection strength was generally reduced in TLE patients compared to controls, reflecting widespread synaptic dysfunction beyond isolated epileptogenic regions. By advancing from regional mappings to a network‐level analysis, we identified complex alterations in synaptic network mechanisms, providing a broader perspective on the neurobiological underpinnings of TLE. Specifically, the hubs of abnormal synaptic connectivity detected within the synaptic connectome encompassed key temporolimbic regions, such as the temporal gyrus, hippocampus, parahippocampal gyrus, amygdala, cingulate gyrus, basal ganglia, as well as frontoparietal association cortices—regions frequently implicated in the epileptogenic network.^[^ ^4^, ^52^, ^53^ ^]^ The limbic system, which maintains tight connections with the hippocampus,^[^ ^54^ ^]^ has been shown to play a crucial role in seizure propagation along synaptic pathways. Furthermore, disruptions in subcortical–cortical circuits^[^ ^55^, ^56^ ^]^—which are central to seizure synchronization and propagation across diffuse, long‐range connections—may represent an additional mechanism contributing to the observed network dysfunction. Our findings shed light on the distributed nature of synaptic connectivity impairments in TLE, highlighting network‐level disruptions across spatially distributed structures rather than isolated abnormalities confined to specific epileptogenic zones. This network‐centric view provides a more comprehensive understanding of how synaptic alterations orchestrate the emergence and propagation of epileptic activity across large‐scale brain networks.

The macroscale synaptic network topology alterations observed in TLE likely arise from multiscale pathological mechanisms,^[^ ^57^ ^]^ spanning genetic predispositions, molecular dysregulations, cellular dysfunction, and circuit‐level reorganization. By employing PLS analysis, we found that abnormal synaptic network connection strengths in TLE were significantly associated with the expression of risk genes. Enrichment analyses revealed that downregulated genes correlated with these network changes were strongly linked to synaptic transmission processes, with significant terms such as “postsynapse,” “postsynaptic density,” “asymmetric synapse,” “postsynaptic specialization,” “neuron to neuron synapse,” “glutamatergic synapse,” “modulation of chemical synaptic transmission,” and “regulation of transsynaptic signaling.” These findings underscore the crucial roles of synaptic dysfunction in TLE. Notably, *ROCK2* emerged as the gene with the highest weight among the downregulated genes and was identified as a target gene by various modules of Cytoscape. The brain slices were subjected to immunofluorescent staining, which revealed significantly elevated *ROCK2* protein levels in surgically resected hippocampal tissue from patients with drug‐resistant TLE compared to nonepileptic controls. Together, these results highlight *ROCK2* as a pivotal regulator of synaptic remodeling in TLE, bridging molecular mechanisms with network‐level disruptions and supporting its potential as a therapeutic target.

The *RhoA* signaling pathway and its downstream effector, *ROCK*, are involved in various neurological disorders, including Alzheimer's disease,^[^ ^58^ ^]^ Parkinson's disease,^[^ ^59^ ^]^ spinal cord injury,^[^ ^60^ ^]^ brain ischemia,^[^ ^61^ ^]^ traumatic brain injury,^[^ ^62^ ^]^ and epilepsy.^[^ ^63^ ^]^ In epilepsy models, nonselective *ROCK* inhibitors such as fasudil or Y‐27632 have been shown to improve early survival rates, alleviate cognitive dysfunction, and reduce epileptic discharge, primarily through mechanisms that enhance neuronal survival and promote synaptic reorganization.^[^ ^62^, ^64^, ^65^, ^66^ ^]^ The *ROCK* family consists of two isoforms, *ROCK1* and *ROCK2*. The *ROCK1* is predominantly expressed in nonneuronal tissues such as the liver, testes, and kidneys, while the *ROCK2* is primarily found in the brain and skeletal muscle.^[^ ^67^, ^68^ ^]^ Importantly, studies have documented decreased expression of *RhoA* and *ROCK1* but an increase in *ROCK2* in a rat model of epilepsy,^[^ ^69^ ^]^ suggesting subtype‐specific roles in epilepsy. *ROCK2* is critical for synapse formation and function,^[^ ^70^ ^]^ exhibiting widespread expression in the brain, including the prefrontal cortex^[^ ^71^ ^]^ and hippocampus.^[^ ^70^ ^]^ We observed a specific high expression of *ROCK2* in a mouse model of TLE and validated synaptic loss in the hippocampus and cortex through in vivo micro‐PET imaging and histological analysis. Furthermore, the selective *ROCK2* inhibitor administered across different animal models (including acute, chronic, and drug‐resistant TLE models) partially reversed synaptic and neuronal loss, enhanced network efficiency within the synaptic density connectome, improved seizure symptoms, and enhanced cognitive performance in the mice. Our findings extend previous research, indicating that in TLE, *ROCK2*, rather than *ROCK1*, predominantly influences synaptic reorganization. Additionally, we present a noninvasive in vivo SV2A PET imaging approach to dynamically assess synaptic loss and the therapeutic effects of *ROCK2* inhibitors in TLE.

To elucidate the neurobiological mechanisms by which *ROCK2* contributes to synaptic loss and reorganization, we conducted RNA sequencing on hippocampal tissue from a mouse TLE model. GO analysis revealed the involvement of synapse‐related signaling pathways, while Reactome pathway analysis highlighted significant enrichment of autophagy‐related pathways. Prior studies have identified mitophagy in hippocampal tissue from patients with refractory TLE, marked by colocalization of the autophagosome marker *LC3B* and the mitochondrial marker *TOMM20*.^[^ ^72^ ^]^ *ROCK2*, a serine/threonine kinase, negatively regulates Parkin‐mediated mitophagy via the PTEN–Akt–HK2 signaling axis.^[^ ^73^ ^]^ Pharmacological inhibition of *ROCK2* promotes HK2 phosphorylation and mitochondrial translocation, which facilitates Parkin recruitment and subsequent mitophagy activation.^[^ ^59^ ^]^ Our results demonstrated dysregulated mitophagy in TLE mice and epileptic cells, characterized by impaired mitochondrial clearance and dysfunction, which were notably absent in the group treated with *ROCK2* inhibitor. Treatment with the *ROCK2*‐specific inhibitor restored mitophagy, reduced the accumulation of damaged mitochondria, and alleviated neurodegeneration and synaptic loss. While the neuroprotective role of *ROCK2* inhibition in epilepsy has been previously acknowledged,^[^ ^62^ ^]^ this study is the first to establish a direct link between *ROCK2* inhibition, enhanced mitophagy, and synapse protection. By elucidating the mechanisms of action underlying these effects, we can better leverage *ROCK2* inhibitors for targeted therapies in epilepsy. These mechanistic insights, combined with the pharmacological advantages of *ROCK2*‐specific inhibition, support the therapeutic potential of belumosudil. Unlike nonselective *ROCK* inhibitors such as fasudil and ripasudil that target both *ROCK1* and *ROCK2*, belumosudil demonstrates selective inhibition of *ROCK2*. The nonselective inhibitors are associated with systemic adverse effects including hypotension, lymphopenia, cerebral hemorrhage, and hepatic or renal dysfunction, effects that are largely attributed to *ROCK1* inhibition.^[^ ^74^, ^75^, ^76^ ^]^ Clinically, belumosudil has been approved in China for chronic graft‐versus‐host disease, demonstrating a favorable safety and tolerability profile, with pneumonia being the most frequently reported adverse event (≈7%).^[^ ^77^ ^]^ Pharmacokinetically, it exhibits a mean elimination half‐life of ≈19 h and a clearance of 9.83 L h^−1^, supporting its suitability for chronic administration in CNS disorders.^[^ ^78^ ^]^ Although predictions suggest moderate BBB permeability, direct experimental evidence of CNS penetrance remains limited. Further evaluation is needed to optimize dosing, confirm CNS bioavailability, and assess long‐term safety in neurological contexts. Given its selectivity for *ROCK2*, established in vivo efficacy, and pharmacokinetic stability, belumosudil represents a promising candidate for modulating aberrant synaptic networks in epilepsy. These findings warrant further preclinical and clinical studies to validate its CNS applicability and translational potential.

Despite these promising findings, several limitations must be acknowledged. First, the transcriptomic data used in this study were derived from a limited number of postmortem brains and a previously published dataset.^[^ ^40^ ^]^ Although these data provide high spatial resolution and disease‐relevant molecular insights, they may not fully reflect the heterogeneity of gene expression across broader patient populations, particularly in living individuals at different disease stages. Future studies incorporating larger‐scale, patient‐specific transcriptomic data, particularly single‐cell or spatial transcriptomics from surgical epilepsy specimens, will be important to further validate and refine our findings. Second, this was a single‐center study, which may restrict the generalizability of our findings to broader clinical populations. Third, although we employed three distinct models of TLE, including acute, chronic, and drug‐resistant phases, these mouse models cannot fully replicate the complexity and variability observed in human TLE. To bridge the translational gap, we conducted preliminary biosafety evaluations to assess the potential clinical applicability of *ROCK2* inhibition. However, additional long‐term studies are necessary to confirm the sustained efficacy and safety of this therapeutic approach in more clinically relevant settings. Fourth, while our results demonstrated therapeutic benefits of *ROCK2* inhibition during both acute and chronic (two months) phases of epilepsy, longer‐term follow‐up studies are needed to evaluate the durability of these effects and monitor potential adverse outcomes associated with extended treatment. Finally, our results are specific to temporal lobe epilepsy, and further research is warranted to elucidate the unique synaptic network alterations and risk gene expressions in other types of epilepsy.

In summary, this study highlights the critical interplay between in vivo synaptic network topological alterations and the downregulation of the spatial expression profiles of risk genes, identifying *ROCK2* as a key therapeutic target for modulating synaptic circuits in TLE. Through a combination of bioinformatics analysis and validation in animal models, we demonstrated that *ROCK2*‐specific inhibitors effectively mitigate epileptic seizures by enhancing mitochondrial autophagy and promoting synaptic remodeling. These findings not only hold promise for the development of targeted therapeutics but also provide valuable insights into the pathophysiological mechanisms underlying molecular network abnormalities in epilepsy. By integrating perspectives from genes, molecular pathways, and neural circuits, this study offers a more comprehensive understanding of the complex mechanisms driving epileptic brain network disorders.

## 4 Experimental Section

### Study Design

In the human experiment, one PET cohort and two independent transcriptomic cohorts were integrated to investigate the associations between alterations in the synaptic density network of patients with TLE and risk gene expression, identifying *ROCK2* as a pivotal gene. The study was conducted following the guidelines for human subject research and was approved by the Ethics Committee of Xiangya Hospital, Central South University (Approval No. 202106134). Written informed consent was obtained from all participants or their authorized representatives.

In the mouse experiment, pilocarpine‐induced seizure models were established and the mice were divided into two groups: one treated with a *ROCK2*‐specific inhibitor and the other given a saline solution. Behavioral analyses, cognitive assessments, in vivo micro‐PET imaging, and histological evaluations were conducted to validate the efficacy of *ROCK2* treatment. Additionally, through mRNA sequencing of the mouse hippocampus and scanning TEM, the molecular mechanisms by which *ROCK2* inhibitors promoted synaptic loss and facilitated synaptic network remodeling were elucidated by enhancing mitophagy. All animal experiments were approved by the Animal Care and Use Committee of Central South University (Approval No. CSU‐2022‐0535).

### Human Studies

The PET cohort included 25 patients with TLE and 23 healthy controls. All participants underwent [^18^F]SynVesT‐1 PET scanning between November 2021 and March 2024. Inclusion criteria for patients were: 1) medically refractory TLE,^[^ ^2^, ^79^ ^]^ 2) ongoing antiepileptic treatment at the time of recruitment, and 3) undergoing the [^18^F]SynVesT‐1 PET scan. Exclusion criteria included: 1) neurologic disorders other than epilepsy, 2) prior neurosurgical interventions, and 3) use of levetiracetam or brivaracetam treatment.^[^ ^33^ ^]^ The diagnosis and lateralization of TLE were confirmed based on seizure semiology, neurologic examination, neuroimaging findings (3‐T MRI, [^18^F]FDG PET), video electroencephalography, and neuropsychological assessment. Healthy controls were matched for demographic characteristics with TLE patients, excluding those with neurological or psychiatric disorders, or drug addiction.

Transcriptomic analysis was conducted using hippocampal dentate gyrus brain tissue obtained from six TLE patients undergoing standard anterior temporal lobectomy at Xiangya Hospital and six healthy postmortem donors at Xiangya School of Medicine, as previously reported.^[^ ^40^ ^]^ Control donors were validated to have no history of neuropsychiatric disorders based on clinical and histopathological evaluations.

### Structural MRI Acquisition

All participants underwent high‐resolution MRI imaging on a 3T Siemens MAGNETOM Trio, a Tim system. 3D T1‐weighted anatomical images were acquired using the magnetization‐prepared rapid acquisition gradient echo (MPRAGE) sequence for coregistration with PET images. The parameters for the MPRAGE sequence were as follows: repetition time = 2300 ms, echo time = 3 ms, field of view (FOV) = 256 × 256 mm^2^, slice thickness = 1.0 mm, 176 sagittal slices, and voxel size = 1.0 × 1.0 × 1.0 mm^3^.

### Clinical PET Image Acquisition and Processing

[^18^F]SynVesT‐1 PET imaging was performed using the Discovery Elite PET/CT scanner (GE Healthcare). [^18^F]SynVesT‐1 was synthesized as described previously and administered intravenously at a dose of 3.7 MBq kg^−1^ over 1 min.^[^ ^80^ ^]^ All patients were seizure‐free in the 24 h before PET scanning. Scans were taken 60 min postinjection, with a low‐dose CT scan obtained simultaneously for attenuation correction. The parameters for the PET/CT scan were as follows: FOV = 350 mm × 350 mm^2^, slice thickness = 3.75 mm, scanning range covered the entire brain with 47 slices, and PET reconstruction implemented via an ordered subset expectation maximization algorithm. The iterative reconstruction process used a matrix size of 256 × 256, a slice thickness of 3.75 mm, two iterations, and 24 subsets. During the scanning procedure, participants were instructed to minimize head movement as much as possible.

Image preprocessing was performed using the SPM12 toolkit (https://www.fil.ion.ucl.ac.uk/spm/) implemented in MATLAB R2018b. Individual [^18^F]SynVesT‐1 PET scans were initially coregistered with their corresponding T1‐weighted structural MRI images and then spatially normalized into standard stereotactic Montreal Neurological Institute space and resliced to 2 × 2 × 2 mm. The normalized PET images were spatially smoothed using an 8 mm full‐width at half‐maximum Gaussian kernel. After processing, the SUVR for each voxel was calculated for PET images of all participants, with the centrum semiovale as the reference region.^[^ ^51^, ^81^ ^]^

### Synaptic Density Similarity Network Construction

Consistent with the prior methodology^[^ ^82^ ^]^ and leveraging the high‐resolution parcellation of the Brainnetome Atlas,^[^ ^83^ ^]^ individualized synaptic density networks were constructed based on its 246 anatomically defined ROIs. Kernel Density Estimation was employed to estimate probability density functions for the [^18^F]SynVesT‐1 uptake distribution in each ROI with automatically determined bandwidths and 2^9^ sampling points as suggested by the previous study.^[^ ^84^ ^]^ The synaptic density connections were derived using the symmetric Kullback–Leibler divergence, mathematically represented as follows

(1) $$D_{\mathrm{KL}}\left(P,Q\right)=\sum_{i=1}^{n}\;\left(P\left(i\right)\log\frac{P\left(i\right)}{Q\left(i\right)}+Q\left(i\right)\log\frac{Q\left(i\right)}{P\left(i\right)}\right)$$

here, *P* and *Q* represent the probability distribution functions of voxel intensities in a pair of ROIs, and *n* denotes the number of sample points. To constrain the measure within a range of 0–1, the synaptic density connectivity strength between pairwise ROIs was transformed using KL divergence as follows

(2) $$\mathrm{KLS}\left(P,Q\right)=e^{-D_{\mathrm{KL}}\left(P,Q\right)}$$

From the KL divergence calculations, a 246 × 246 adjacency matrix was obtained that represented the pairwise synaptic density connectivity. This adjacency matrix was utilized to construct individual synaptic density connectome (no thresholding) based on PET images of all participants, facilitating further network analysis.

### Network Properties Analysis

Using scripts provided by the Brain Connectivity Toolbox,^[^ ^85^ ^]^ standard formulas were employed to calculate the nodal connectivity strength within the synaptic density connectome in controls and patients with TLE. Nodal connectivity strength quantified the degree to which brain regions were interconnected to all other brain regions. It was one of the most widely used graph‐theoretical parameters for describing the topological properties of complex networks. Age and sex were included as covariates to minimize the effects of confounding factors.

### Transcriptome Sequencing

Transcriptome sequencing was conducted by CapitalBio Technology Co., Ltd. (Beijing, China) according to standard protocols. Total RNA was extracted from hippocampal dentate gyrus tissue, and mRNA was purified using oligo (dT) beads. RNA integrity and quality were assessed using the Agilent 2100 Bioanalyzer (Agilent Technologies, Santa Clara, CA, USA), ensuring high RNA integrity numbers for all samples. Libraries were prepared using the TruSeq Stranded mRNA LT Sample Prep Kit (Illumina, San Diego, CA, USA) according to the manufacturer's instructions, and sequencing was performed on the Illumina HiSeq X Ten platform in paired‐end mode. The results of raw data files in FASTQ format underwent quality control using Trimmomatic^[^ ^86^ ^]^ to remove low quality reads and adapter sequences. Clean reads were then aligned to the human reference genome using HISAT2^[^ ^87^ ^]^ with default parameters.

### Gene Expression Analysis

The AHBA (https://human.brain‐map.org) was a publicly available transcriptional atlas containing gene expression data measured using microarrays, sampled from hundreds of histologically validated neuroanatomical structures across six postmortem human brains (age: 42.50 ± 13.38 years; male/female ratio: 5/1).^[^ ^88^ ^]^ A total of 58 692 microarray mRNA probes were utilized for transcriptome analysis of brain tissue samples in this dataset, which included data from the left hemisphere only (four subjects) and both left and right hemispheres (two subjects).

AHBA transcriptomic data were preprocessed using the abagen toolbox (https://github.com/netneurolab/abagen), following the pipeline described by Arnatkeviciute et al.^[^ ^89^ ^]^ First, the microarray transcriptome data were mapped to the 246 brain regions identified by the BN Atlas,^[^ ^90^, ^91^ ^]^ assigning expression values from tissue samples nearest to the centroid for unmatched regions in the atlas. Second, the differential stability strategy was employed to condense the redundant probes for each gene by calculating and averaging the Spearman correlation coefficient for each value of probe microarray expression among subjects and finally retaining only the probe with the highest correlation coefficient. Additionally, intensity‐based filtering was applied to remove probes with intensity less than 50% above the background noise in all tissue samples. To mitigate potential discrepancies in expression values between donors caused by “batch effects,” the AHBA microarray data underwent several normalization processes but still showed some disparities among donors. A scaled robust sigmoid function was further applied to normalize the microarray gene expression data to mitigate donor‐specific effects.

In performing comparative transcriptome analysis, genes that were significantly differentially expressed in the hippocampal dentate gyrus in TLE (*p* < 0.05, FDR corrected) were linked with data from a recently published transcriptional study^[^ ^41^ ^]^ on a large TLE cohort (*n* = 161) to the gene expression profile by the AHBA, ultimately mapping TLE‐related transcriptome data to the BN Atlas to obtain a topographical gene expression matrix (246 regions × 5451 genes).

### Partial Least Squares Regression Analysis

The correlation between the transcriptional activity of all 5451 genes and changes in nodal connectivity strength of the synaptic density network (*t‐*values from 246 cortical areas) was examined using PLS regression.^[^ ^92^ ^]^ In this regression analysis, gene expression levels (predictor variables) were used to forecast changes in nodal connectivity strength of the synaptic density network (response variables). *PLS1* represented a linear combination of the gene expression that correlated most strongly with changes in nodal connectivity strength of the synaptic density network. The *PLS1* score was then normalized to a *z*‐score value using a bootstrapping approach.^[^ ^39^ ^]^ The relationship between gene expression and changes in nodal connectivity strength of the synaptic density network was assessed using permutation testing, with 5000 randomized permutations of the response variable.^[^ ^93^ ^]^

### Functional Enrichment and Key Gene Identification

Functional enrichment analysis was conducted using Metascape (https://metascape.org/gp/index.html) to identify biological processes and pathways associated with genes exhibiting weight z > 3 or < −3 in the PLS analysis.^[^ ^94^ ^]^ Enrichment entries were found in the GO and DisGeNET databases. The enrichment analysis employed all genes in the genome as the enrichment background, and *p‐*values were calculated using a cumulative hypergeometric distribution and adjusted for multiple comparisons using Benjamini–Hochberg FDR correction (*p* < 0.01). For the hierarchical clustering of gene entries, kappa scores were used as a similarity indicator, with entries considered to be in the same class if their similarity was greater than 0.3; the most significant entry within the class was chosen to represent the class.

To further explore interactions among enriched genes, a gene interaction network was constructed using GeneMANIA.^[^ ^95^ ^]^ The resulting network was analyzed using the MCODE plugin in Cytoscape, which identified highly interconnected clusters (modules) within the gene–gene network.^[^ ^96^ ^]^ MCODE parameters were configured as follows: node score cutoff = 0.2, maximum depth = 100, and *K*‐core = 2, ensuring robust identification of densely connected modules.^[^ ^97^ ^]^ Two additional Cytoscape plug‐ins, CytoHubba^[^ ^98^ ^]^ and CytoNCA,^[^ ^99^ ^]^ were employed to implement multiple algorithms for hub gene detection, further refining the analysis of key regulatory genes within the network.

### KA‐Induced Hippocampal Neuronal Model

Primary mouse hippocampal neurons were prepared following previously established protocols.^[^ ^100^ ^]^ Briefly, embryonic mice aged 18 days were procured, and the hippocampus was digested with 2 mg mL^−1^ papain (Worthington, LS003126) containing 10 U mL^−1^ DNase I at 37 °C for 20 min. Dissociated cells were resuspended in Neurobasal‐A medium (Gibco, 10888022) supplemented with 2% B‐27 (Gibco, 17504044), 0.5 mm GlutaMAX (Gibco, 35050061), and 1% penicillin–streptomycin. Cells were plated at 5 × 10⁴ cells cm^−^^2^ on poly‐l‐lysine‐coated (1 µg cm^−^^2^, Sigma P4707) culture vessels. At DIV 10, neurons were treated with 50 µm kainic acid (MedChemExpress, HY‐N2309) in Mg2+‐free *N*‐(2‐hydroxyethyl)piperazine‐29‐(2‐ethane‐sulfonic acid)‐buffered saline for 30 min, followed by three washes and 24 h recovery in a conditioned medium. Following KA treatment, a neuronal conditioned medium was collected for downstream analyses.

### Animals

Male C57BL/6 mice, aged 7 weeks, were obtained from the Department of Laboratory Animals at Central South University. The mice were housed individually under standard environmental conditions (12 h light–dark cycle) with ad libitum access to food and water.

### Pilocarpine‐Induced Seizure Model

The rodent model of acute epilepsy was established by administering belumosudil through intragastric administration at a daily dosage of 200 mg kg^−1^ body weight for 7 consecutive days to the *ROCK2* group, while the EP group received normal saline. One day after the last treatment, pilocarpine (300 mg kg^−1^, i.p., Sigma, USA) was injected into both groups to induce status epilepticus. This was preceded by racemic hyoscyamine hydrochloride (1 mg kg^−1^, i.p., Department of Pharmacy, Xiangya Hospital, Central South University), administered 30 min prior to the pilocarpine injection. Seizure severity was assessed using the modified Racine scale.^[^ ^101^ ^]^ In addition to the construction of the acute phase epilepsy model, additional experiments were conducted to monitor mouse behavior beginning with a single injection of pilocarpine, assessing the severity of acute seizures at the 10th, 20th, 40th, and 60th min. Diazepam was administered to terminate the seizures, and the mice were used in behavioral experiments, PET imaging, and immunohistochemistry.

For chronic seizure induction, the EP and *ROCK2* groups were initially established using the pilocarpine‐induced seizure model. The success of the chronic epileptic models was determined through behavioral observations of spontaneous seizures. On day 60 of pilocarpine injection, once all epileptic mice had transitioned into the chronic phase, the *ROCK2* group was started on 7 consecutive days of daily belumosudil treatment, while the EP group was treated with saline. Subsequently, behavioral experiments and PET imaging were conducted following the completion of drug administration. After the behavioral experiments, the mice were continuously monitored for one week, during which the number of spontaneous seizures per day was recorded. On the final day, the mice were euthanized, and the hippocampi were collected for further analysis. To establish a drug‐resistant epilepsy mouse model, a portion of the epileptic mice that had transitioned into the chronic phase were treated with carbamazepine (40 mg kg^−1^ per day, i.p., Novartis Biosciences, Brazil) through daily injections for 14 days.^[^ ^102^, ^103^ ^]^ If the number and duration of seizures decreased by more than 50% after treatment, it was judged to be sensitive to carbamazepine treatment. If the number and duration of seizures were reduced by less than 50%, treatment with phenytoin sodium (75 mg kg^−1^ per day, i.p., Novartis Biosciences, Brazil) was continued daily for 14 days. If the reduction in the number and duration of seizures was still less than 50%, it was considered to be drug‐resistant to the two drugs and classified as a drug‐resistant epilepsy mouse model.

To replicate the principal characteristics of human TLE, the mice were randomly assigned to two groups following a 7 day acclimatization period for the acute and chronic phases of epilepsy: 1) epileptic mice treated with saline solution (the EP group), 2) epileptic mice treated with *ROCK2* inhibitor belumosudil (the *ROCK2* group).

### Quantitative Real‐Time PCR Analysis

Total RNA was extracted using Trizol reagent, and reverse transcribed with a reverse transcription kit (*n* = 6).^[^ ^104^ ^]^ Then, quantitative real‐time PCR analysis was performed using a qPCR kit according to the manufacturer's instructions. The primers used *ROCK2* amplification were as follows: forward primer: 5′‐GCAGAAGTGGGTTAGTCGGTTG‐3′; reverse primer: 5′‐GGCAGTTAGCTAGGTTTGTTTGG‐3′.

### Assessment of Behavioral Changes

Behavioral changes in epileptic mice were evaluated and scored using the modified Racine scale (91): 0, no response; 1, immobilization and facial movement; 2, forelimb and or tail extension, rigid posture; 3, repetitive movements, head bobbing; 4, bilateral forelimb clonus with rearing; 5, continuous rearing and falling; 6, severe tonic–clonic convulsions and hindlimb extension; and 7, death. A score of 6 represented status epilepticus, defined as tonic–clonic convulsions lasting for more than 30 min without intermediate recovery.

To evaluate the effect of belumosudil on seizure frequency, both the EP and *ROCK2* groups were monitored during the chronic stage of epilepsy. Continuous video recording was conducted for one week, and the daily seizure count for each mouse was analyzed retrospectively by trained observers blinded to the experimental groups.

### Assessment of Cognitive Function

To assess the potential effect of belumosudil on cognitive function in TLE mice, two widely used behavioral paradigms—the novel object recognition test and the Morris water maze test—were conducted.

The novel object recognition test was a commonly employed behavioral experiment that investigated short‐term recognition memory. On the first day of the experiment, the mice were acclimatized to their surroundings. They were then introduced to two identical objects and allowed 5 min to explore. After 24 h, one of the objects was replaced with a novel object, and the mice were given an additional 5 min trial for exploration. The investigation time of novel object (*N*) and familiar object (*F*) were meticulously recorded. The discrimination index was calculated using the formula: (*N* − *F*)/(*N* + *F*) × 100%.

The Morris water maze test assessed learning ability and spatial memory in TLE mice. A water maze pool, with a diameter of 120 cm, was filled with water maintained at room temperature. During training trials, mice were required to locate the hidden platform (10 cm in diameter) positioned in the center of one quadrant, submerged 1 cm below the water surface, over the course of four consecutive days. Both group of mice had three consecutive daily training trials for 4 days, with an intertrial interval of at least 1 h. The escape latency (s) required to find the platform was analyzed for each trial. For probe trials, the platform was removed 24 h after the last training trial, and the number of times the mice crossed the previously hidden platform was measured within a 60 s interval.

### Micro‐PET Image Acquisition and Processing

All PET scans were performed using a micro‐PET scanner (Mediso, Hungary). Mice were anesthetized with sodium pentobarbital (30 mg kg^−1^, i.p.) prior to the intravenous injection of ≈8 MBq of [^18^F]SynVesT‐1 via the tail vein. Following tracer administration, a static micro‐PET scan (30 min) was acquired during the period 30–60 min postinjection. PET image reconstruction was performed using 3D Tera‐Tomo algorithms with Monte‐Carlo‐based estimation for scatter and attenuation correction.

Image processing was conducted using PMOD software. For the standardized delineation of target brain regions, the T1 MRI template from the Ma–Benveniste–Mirrione atlas,^[^ ^45^ ^]^ as previously described^[^ ^46^ ^]^ was utilized. The animal‐specific CT datasets were spatially normalized to the MRI dataset from this atlas, and the corresponding transformation matrices were employed to normalize the PET datasets according to the Mirrione matrix. All transformations were executed using a rigid matching algorithm as implemented in PMOD. The radioactivity concentrations extracted from the hippocampus and cortex were expressed in SUV after normalization for the injected dose and the body weight of the animals. The SUV measurements were derived from the 30–60 min period of the scan during which the time‐activity curves were observed to be at a steady state.

### Network Analysis

Network analysis was performed in Matlab (MathWorks, 2021b) using the Brain Connectivity Toolbox (BCT) as previously described,^[^ ^85^, ^105^ ^]^ leveraging generalized Louvain modularity^[^ ^106^ ^]^ and hierarchical consensus clustering.^[^ ^107^ ^]^ The analysis code was made publicly available in the CovNet package on GitHub (https://github.com/echumin/CovNet).

Covariance networks for EP and *ROCK2* groups (*n* = 12 per group) were computed using Pearson correlation of regional *z*‐scored SUV values. This resulted in standardized uptake a region‐by‐region (22 × 22 regions) adjacency matrix for each group, representing the similarity of synaptic density coupling between all region pairs as measured by [^18^F]SynVesT‐1. Three network variants were generated: weighted and unthresholded; weighted and thresholded at *p* < 0.05 correlation significance; and weighted and thresholded at *p* < 0.01 correlation significance. Edge‐level correlation significance was assessed using a random shift null model with 10 000 permutations, independently permuting SUV for each region and disrupting the covariance structure. The distribution of these null networks was then used to estimate permutation *p*‐values for each edge, representing the proportion of times the empirical correlation exceeded the null magnitude, treating positive and negative correlations separately. Thresholded networks were analyzed using BCT to compute the following measures: network density (number of connections), node degree (connections per region), positive and negative node strength (sum of covariance values per region), and clustering coefficient (considering fully connected triangles and the weight and sign of connections).^[^ ^108^ ^]^ Hub regions were identified as the top quartile regions based on network strength (strength‐based) and degree (degree‐based). Strength‐based hubs were estimated from both unthresholded and thresholded networks, whereas degree‐based hubs were specific to thresholded networks.

### Immunohistochemical Staining

Tissues from the brain, kidney, liver, lung, spleen, and heart were postfixed in 4% paraformaldehyde at room temperature overnight, followed by dehydration through a graded series of alcohols and subsequent paraffin embedding. Hippocampal specimens from TLE patients (surgical resection), postmortem controls, and mouse models were processed for SV2A staining. Hippocampal sections from mouse models were further subjected to Nissl or NeuN staining, while other tissues were stained using hematoxylin and eosin following standard protocols. Slides were analyzed under a Pannoramic MIDI microscope, and representative images were randomly captured for each sample.

### Immunofluorescence Staining

Hippocampal sections (10 µm) from TLE surgical specimens and postmortem controls were fixed with 4% PFA for 20 min at 4 °C. After permeabilization with 0.1% Triton X‐100 (3 × 5 min), sections were blocked with 5% normal donkey serum for 1 h at RT. Primary antibody incubation was performed using rabbit anti‐*ROCK2* (1:100) at 4 °C for 16 h. After washing, sections were incubated with Alexa Fluor 488‐conjugated donkey anti‐rabbit IgG (1:500) for 2 h at RT in the dark. Nuclei were stained with DAPI (1 µg mL^−1^) for 5 min. Sections were mounted with ProLong Diamond Antifade Mountant and imaged using a Zeiss 710 confocal microscope.

### High‐Throughput RNA Sequencing

Quality control of RNA‐seq data from hippocampal tissue samples of mice was performed using Trim Galore, followed by mapping to the hg19 genome using Hisat2 with default parameters. The read counts for each gene were calculated using featureCounts with the parameters ‐s 2 and ‐M. The expression of differentially expressed genes between the EP group and the *ROCK2* group was subsequently obtained.

### Differential Expression Analysis and Enrichment Analysis

Differential gene expression was analyzed using DESeq (R package). DEGs were identified based on the criteria of |log2 fold change| ≥ 1.5 and *p* ≤ 0.05 for statistical significance.

DEGs were represented using graphs to calculate the number of upregulated and downregulated DEGs in each comparison group. Volcano plots illustrating the gene distribution of DEGs were created. Two‐way clustering analysis of the union and samples of DEGs across all comparison groups was performed. GO enrichment analysis and Reactome pathway analysis were conducted with the GO and Reactome database using Metascape (https://metascape.org/gp/index.html), focusing on significantly enriched pathways with *p* < 0.05.

### Molecular Docking

Protein–protein docking simulations were performed to evaluate interactions between *LC3* and *ROCK2* proteins using HADDOCK.^[^ ^109^ ^]^ Residue information for both proteins was used to define active interaction sites. The docking generated 27 models grouped into 5 clusters, accounting for 13.0% of all refined models. The top 5 clusters were analyzed, and detailed results are provided in Table S4 (Supporting Information).

### Transmission Electron Microscopy

TEM was used to assess mitophagy in KA cells and the hippocampal region. Samples were fixed with 2.5% glutaraldehyde for 2 h at 37 °C, followed by postfixation with 1% osmium tetraoxide for 1 h. The fixed tissues and cells were then dehydrated using a graded ethanol series and embedded in Spurr resin. Ultrathin sections 70 (nm) were prepared and mounted onto nickel grids, followed by staining with uranyl acetate and lead citrate. Images were captured using a TEM (JEM‐1230, JEOL, Japan) and analyzed using ImageJ software to evaluate morphological changes indicative of mitophagy.

### Determination of Mitochondrial Transmembrane Potential (Δ*Ψ*_m_)

Δ*Ψ*_m_ was measured using the JC‐1 assay. Cells were seeded in a 24‐well plate and treated for 48 h according to experimental groups. After treatment, cells were stained with JC‐1 dye for 20 min at 37 °C in the dark. JC‐1 monomers (low Δ*Ψ*_m_) and J‐aggregates (high Δ*Ψ*_m_) were visualized using a fluorescence microscope (DMI16000B, Leica, Germany).

### Lactate Dehydrogenase Assay

The release LDH from damaged cells was measured to evaluate cytotoxicity. Cells were seeded in 96‐well plates at a density of 5 × 10^3^ cells per well (in triplicates) and cocultured with KA cells for 48 h. Following incubation, cell supernatants were collected and processed using a LDH assay kit (Roche, Prague, Czech Republic) according to the manufacturer's protocol. Samples were incubated with LDH substrate for 10 min, and absorbance was measured at 490 nm (with a reference excitation at 630 nm) using an Infinite M200 Pro plate reader (Tecan, Schoeller Instruments, Prague, Czech Republic).

### Prediction of BBB Permeability

Using ACD/Percepta 14.53.0 software, the BBB permeability of the *ROCK2* inhibitor was predicted. Key parameters included: logBB, reflecting the steady‐state ratio of brain to plasma drug concentration; logPS, representing the permeability–surface area product based on capillary transport kinetics; and log(PS × fu, brain), which combined permeability with the unbound fraction in brain tissue to estimate the rate of brain–plasma equilibrium.

### Serological Analysis

To evaluate the biosafety profile of the selective *ROCK2* inhibitor belumosudil on major organs (particularly hepatic and renal systems), serum levels of UA, ALT, AST, CRE, and UREA were quantified. Blood samples were collected from belumosudil‐treated (*ROCK* group) and EP group mice at an endpoint. After 30 min clotting at room temperature, samples were centrifuged at 3000 × *g* for 15 min to isolate serum, which was aliquoted and stored at −80 °C until analysis. Serum biochemical parameters were measured using an automated biochemistry analyzer (Cobas c501, Roche Diagnostics) following manufacturer protocols.

### Statistical Analysis

Data were presented as mean ± standard deviation (SD) unless otherwise specified. Statistical analysis was performed using GraphPad Prism (version 8.0.2). Student's *t*‐test was employed to compare differences between the two groups, while one‐way ANOVA, followed by post‐hoc tests, was applied for multiple groups. Chi‐square (*χ* ^2^) analyses were conducted for categorical variables, and Fisher's exact test was performed to assess survival in the acute phase. The critical threshold for statistical significance was established at *p* < 0.05. The exact value of *n* in the figures and the corresponding replicates were indicated in the figure legends.

## Conflict of Interest

The authors declare no conflict of interest.

## Supporting information



Supporting Information

## Data Availability

The data that support the findings of this study are available on request from the corresponding authors. The data are not publicly available due to privacy or ethical restrictions.
