# Supplementary material for: Targeting ROCK2 to Restore Epileptic Synaptic Networks via Mitophagy Activation: Insights from Translational Imaging of SV2A In Vivo
Source: Adv Sci (Weinh). 2025 Aug 29;12(42):e08161. doi: 10.1002/advs.202508161 (PMC12622553; doi:10.1002/advs.202508161)
Supplement: Supplementary file 1 — Supporting Information [file ADVS-12-e08161-s001.docx]

**Supplementary** **Information for:**

**Targeting ROCK2 to restore epileptic synaptic networks via mitophagy activation: Insights from translational imaging of SV2A in vivo**

**Supplementary Figures**


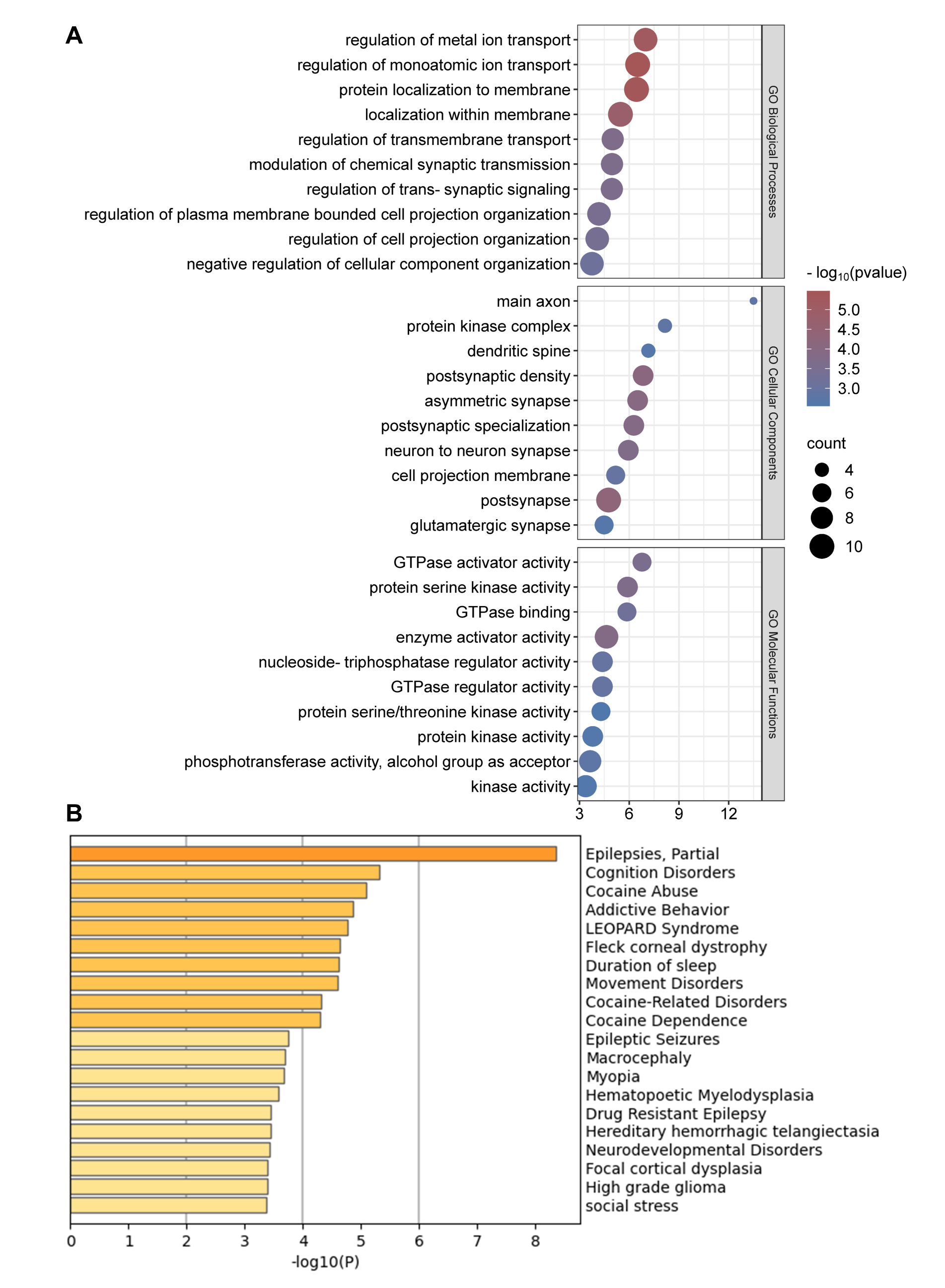


**Fig. S1. Significantly positively weighted (*Z* < -3) and downregulated genes were subjected to functional enrichment analysis.** (A) The top 30 enriched terms in the GO enrichment. (B) The top 20 enriched terms in the ontology category of DisGeNET. Term criteria were as follows: *P* < 0.01, a minimum count of 3, and an enrichment factor > 1.5.


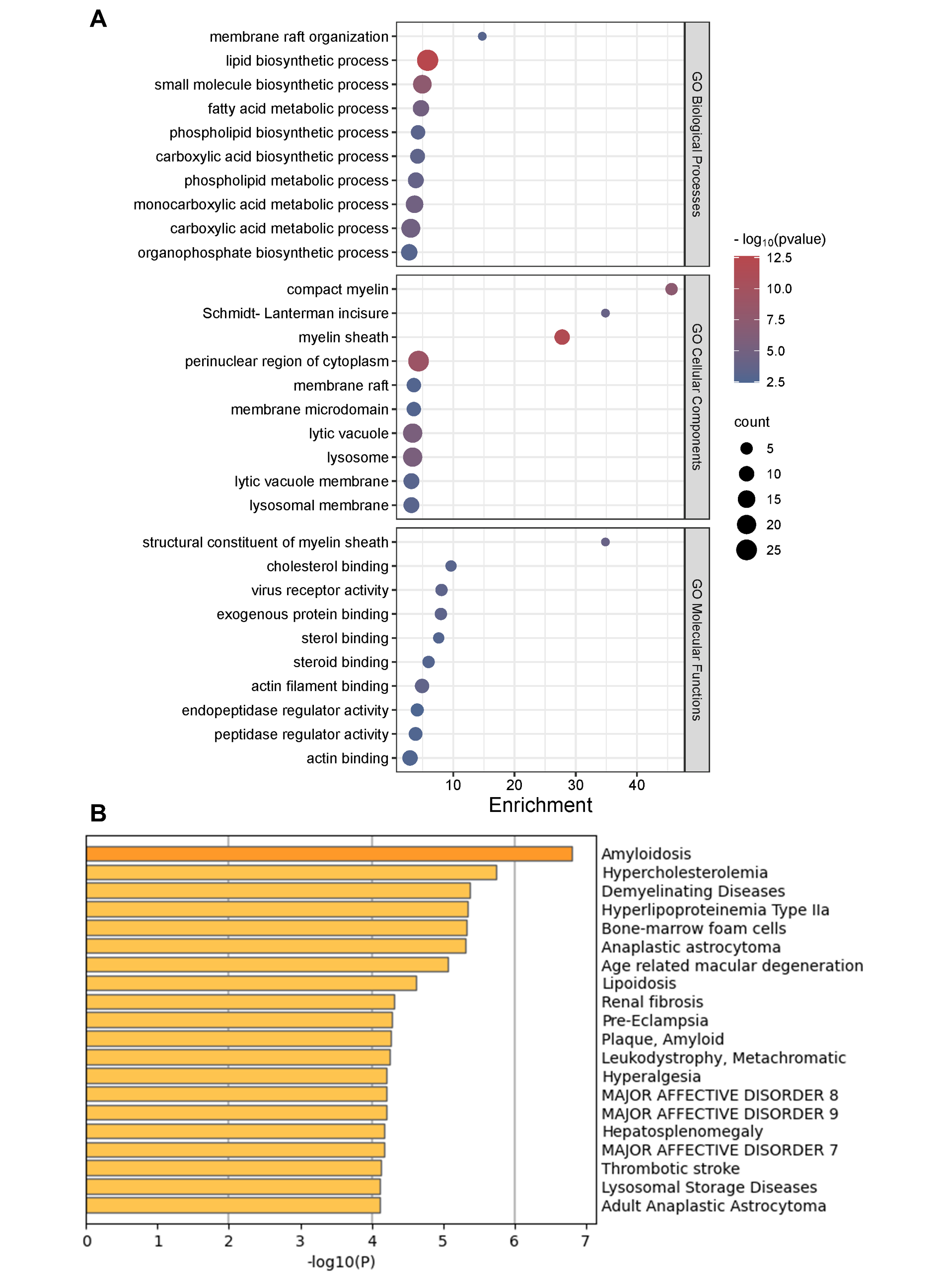


**Fig. S2. Significantly positively weighted (*Z* > 3) and upregulated genes were subjected to functional enrichment analysis.** (A) The top 30 enriched terms in the GO enrichment. (B) The top 20 enriched terms in the ontology category of DisGeNET. Term criteria were as follows: *P* < 0.01, a minimum count of 3, and an enrichment factor > 1.5.


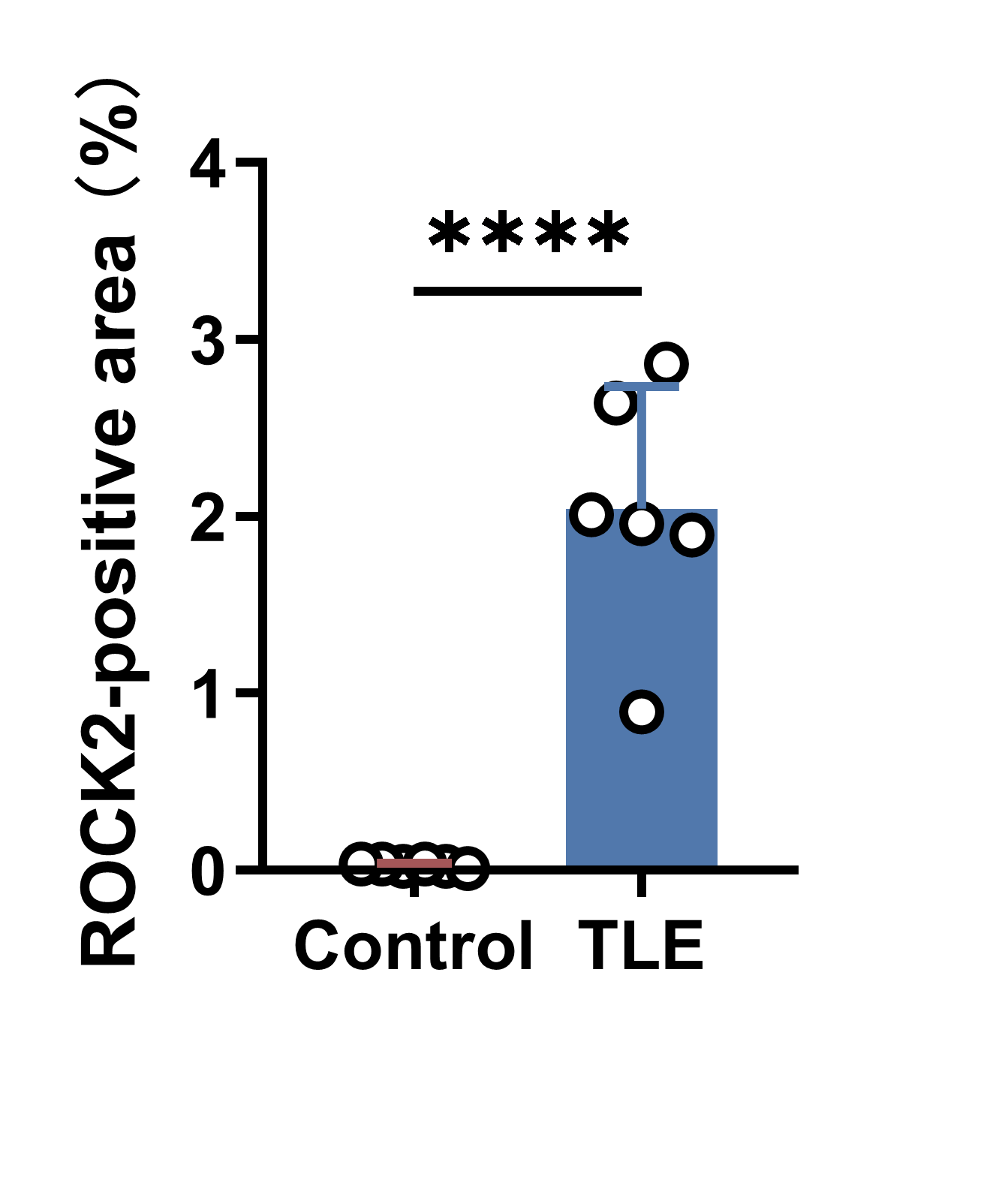


**Fig. S3 Quantitative analysis of ROCK2 immunofluorescence.** The ROCK2-positive area was significantly increased in the brain tissue of patients with TLE compared to postmortem controls. Error bars represent standard deviation (SD). *****P* < 0.0001.


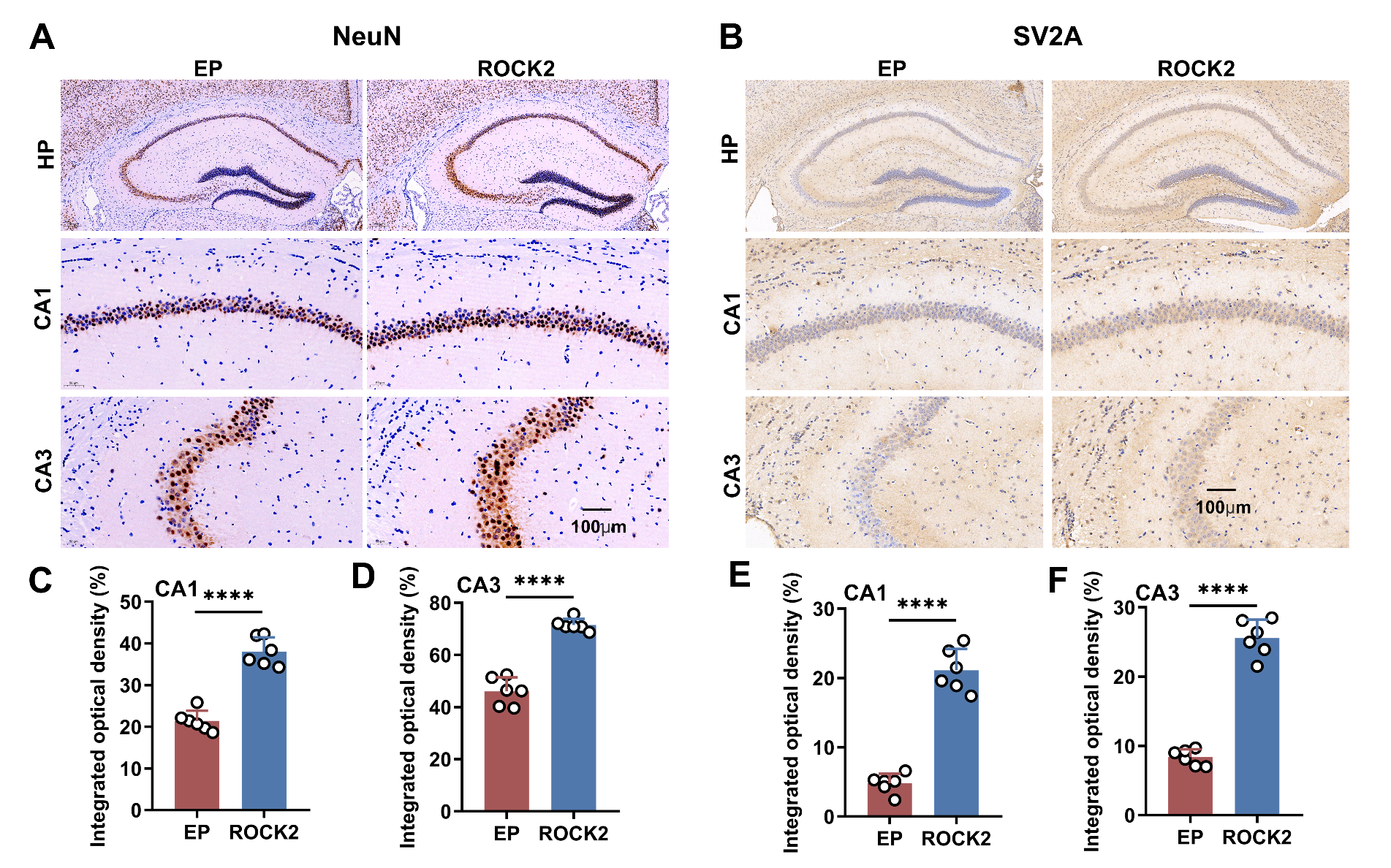


**Fig. S4.** (A, C, and D) Representative images and quantification analysis of NeuN staining in the hippocampal CA1 and CA3 regions of the EP and ROCK2 groups. (B, E, and F) Representative images and quantification analysis of SV2A expression in hippocampal subregions including CA1 and CA3 in the two groups. *****P* < 0.0001.


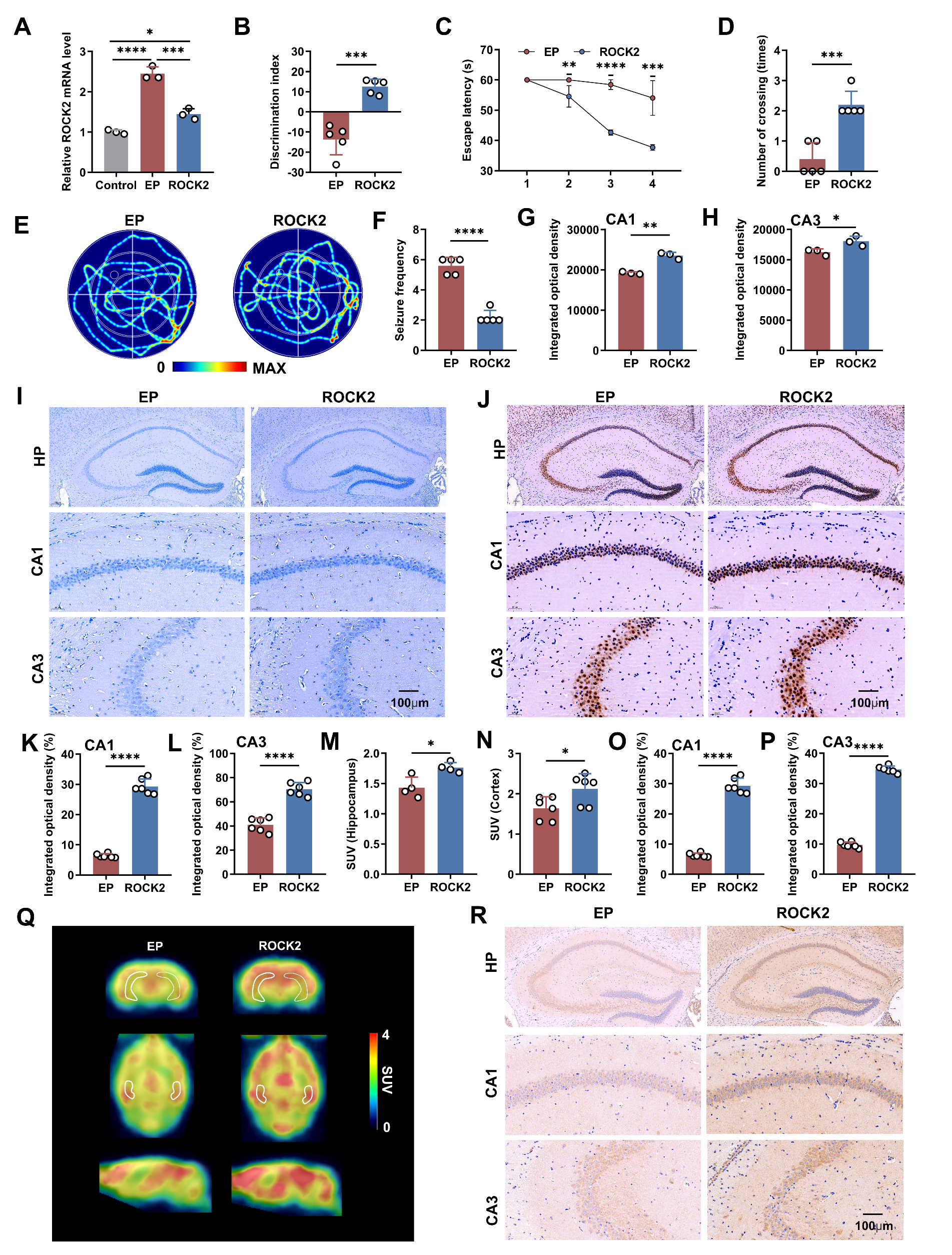


**Fig. S5. Effects of ROCK2 inhibitor on TLE mice in the drug-resistant phase.** (A) The expression levels of ROCK2 in the hippocampal tissues of mice in the control group, EP group, and ROCK2 group. (B) The discrimination indices for NOR tests of mice with TLE. (C) Escape latency of mice to find the emerged platform during the training trial. (D) Number of times mice crossed the target location during the probe test. (E) Swimming traces of mice in the probe trial. (F) The average number of seizures per day in mice. (G, H, and I) Representative images and quantification analysis of Nissl staining in the hippocampal CA1 and CA3 regions of the EP and ROCK2 groups. (J, K, and L) Representative images and quantification analysis of NeuN staining in the hippocampal CA1 and CA3 regions of the two groups. (M, N, and Q) Representative coronal, axial, and sagittal SUV images of [^18^F]SynVesT-1 PET (from 30 to 60 min) in the two groups, and SUV values in the hippocampus and cortex regions. The structures outlined in white are the bilateral hippocampus. (O, P, and R) Representative images and quantification analysis of SV2A expression in hippocampal subregions including CA1 and CA3 in the two groups. **P* < 0.05, ***P* < 0.01, ****P* < 0.001, *****P* < 0.0001.

**
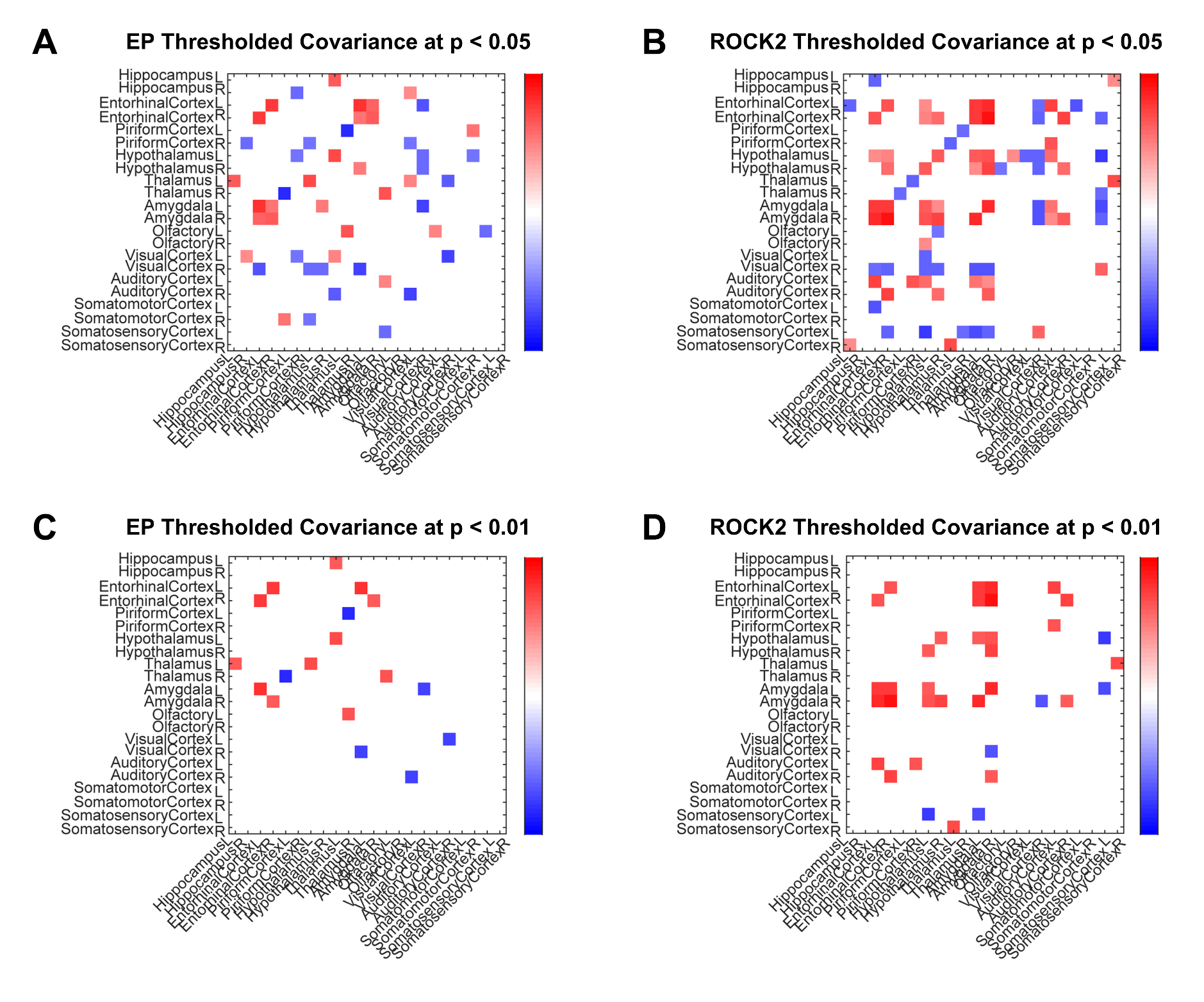
Fig. S6. The synaptic density ([^18^F]SynVesT-1) covariance matrices EP (A, C) and ROCK2 (B, D) groups.** Matrices were thresholded at permutation *P* < 0.05 and *P* < 0.01 correlation significance. Covariance was computed as Pearson correlation of *z*-scored regional standardized uptake value ratio values across animals within each group.

**
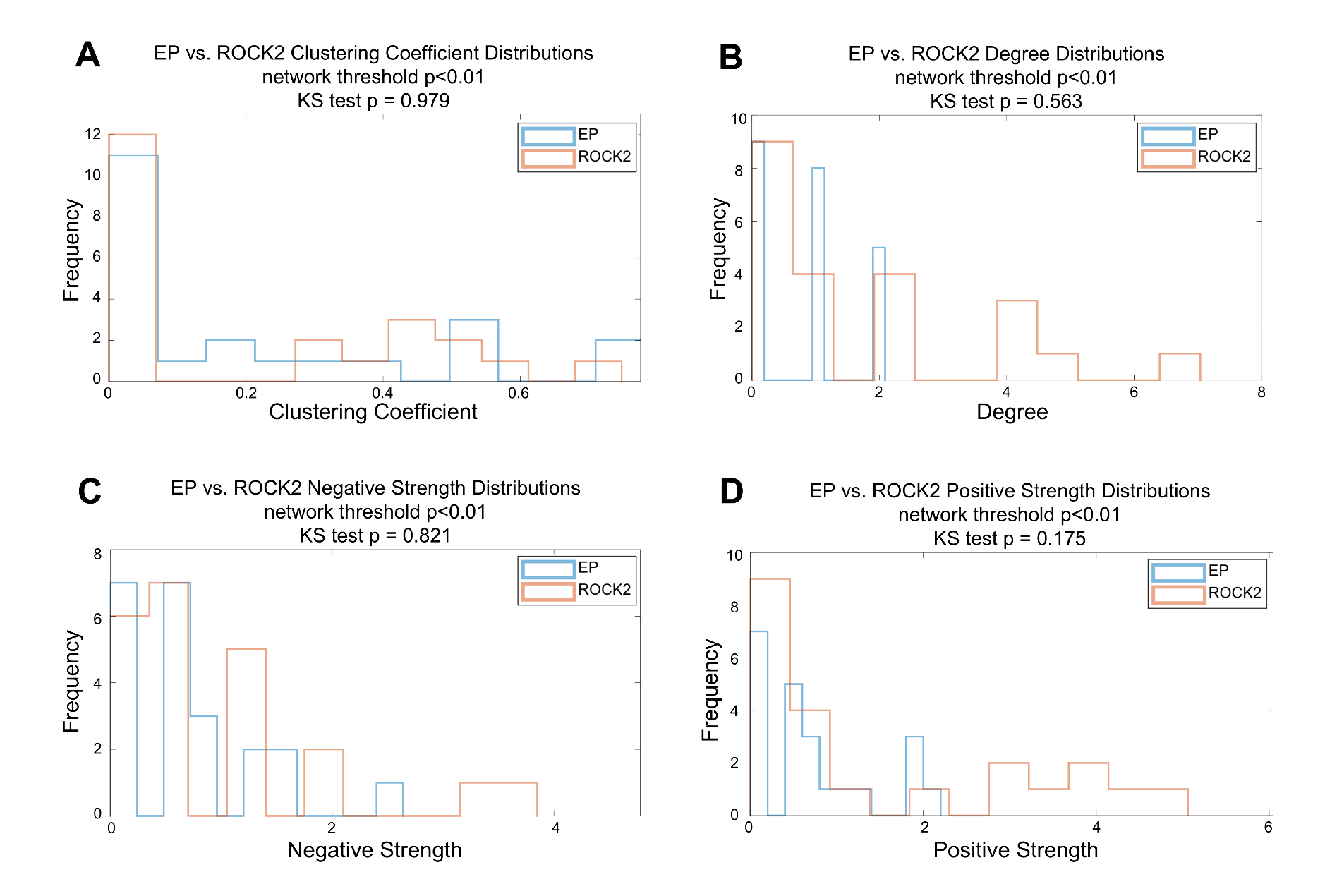
Fig. S7. Distributions of nodal properties of *P* < 0.01 thresholded covariance networks for EP and ROCK2 groups.**


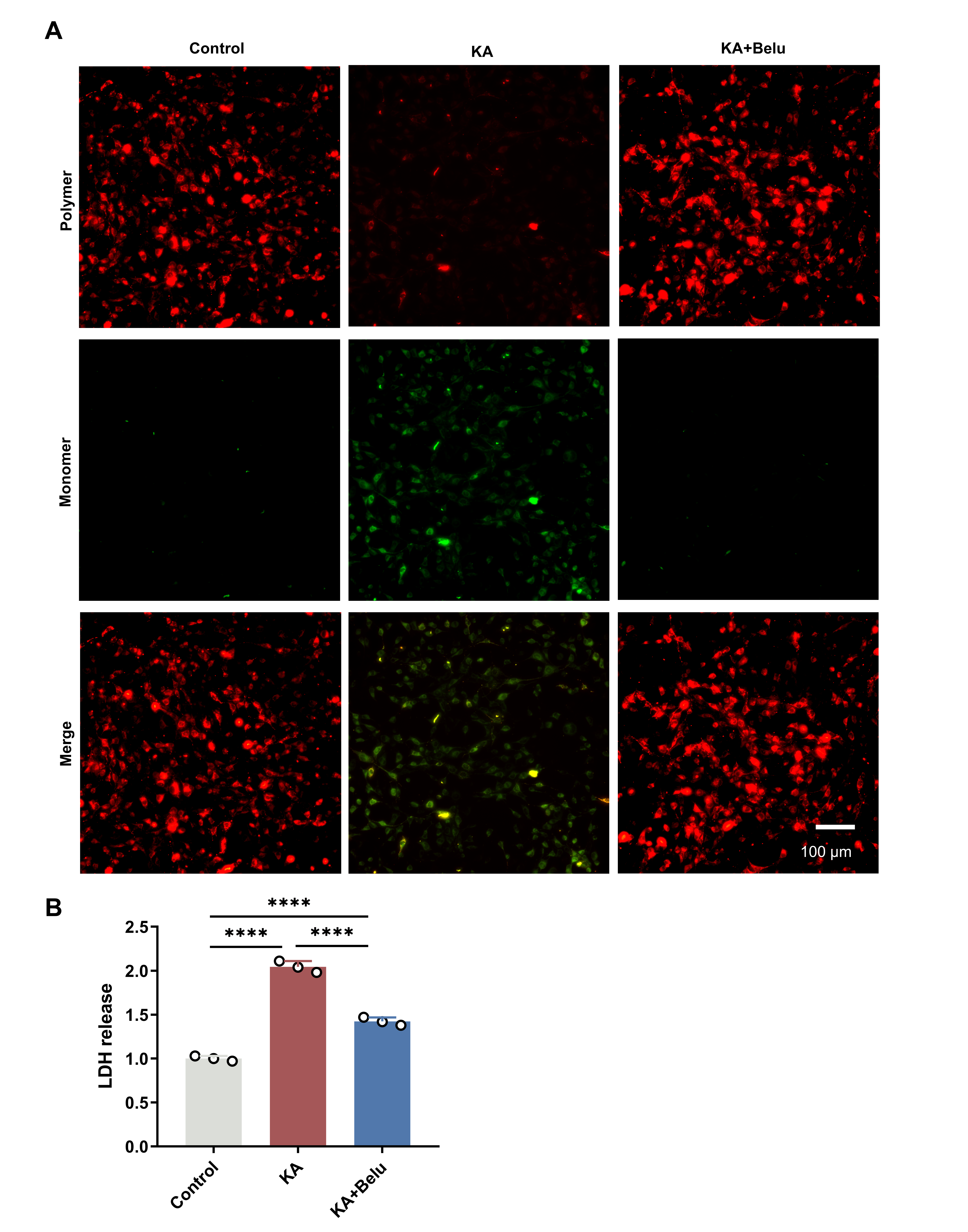


**Fig. S8 Effect of ROCK2 inhibitor on depolarization of mitochondrial membrane potential and cytotoxicity** **in KA cells.** (A) Red JC-1 dimers indicate normal mitochondrial membrane potential. Green JC-1 monomers reflect the depolarization of mitochondrial membrane potential. The red dimers and green monomers co-localized. Experiments were repeated three times each. (B) Cytotoxicity was assessed by LDH assay.


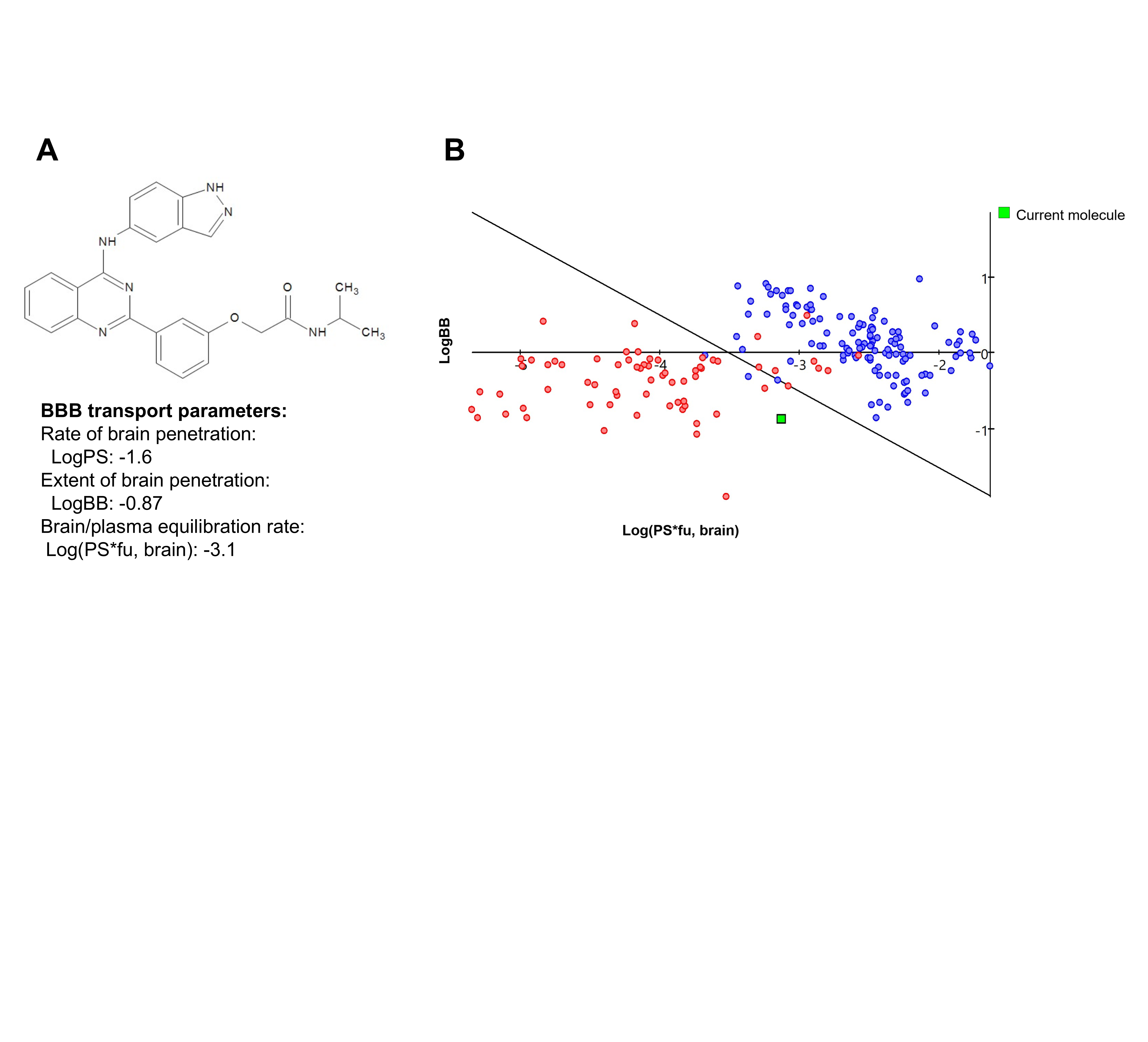


**Fig. S9 The average BBB permeability of ROCK2 inhibitor.** (A) 2D structure of the ROCK2 inhibitor. (B) Scatter plot comparing the BBB permeability of ROCK2 inhibitor with known central nervous system drugs and peripheral drugs, where green dots represent ROCK2 inhibitor, blue dots indicate established central nervous system medications, and red dots denote peripheral-acting drugs.

**
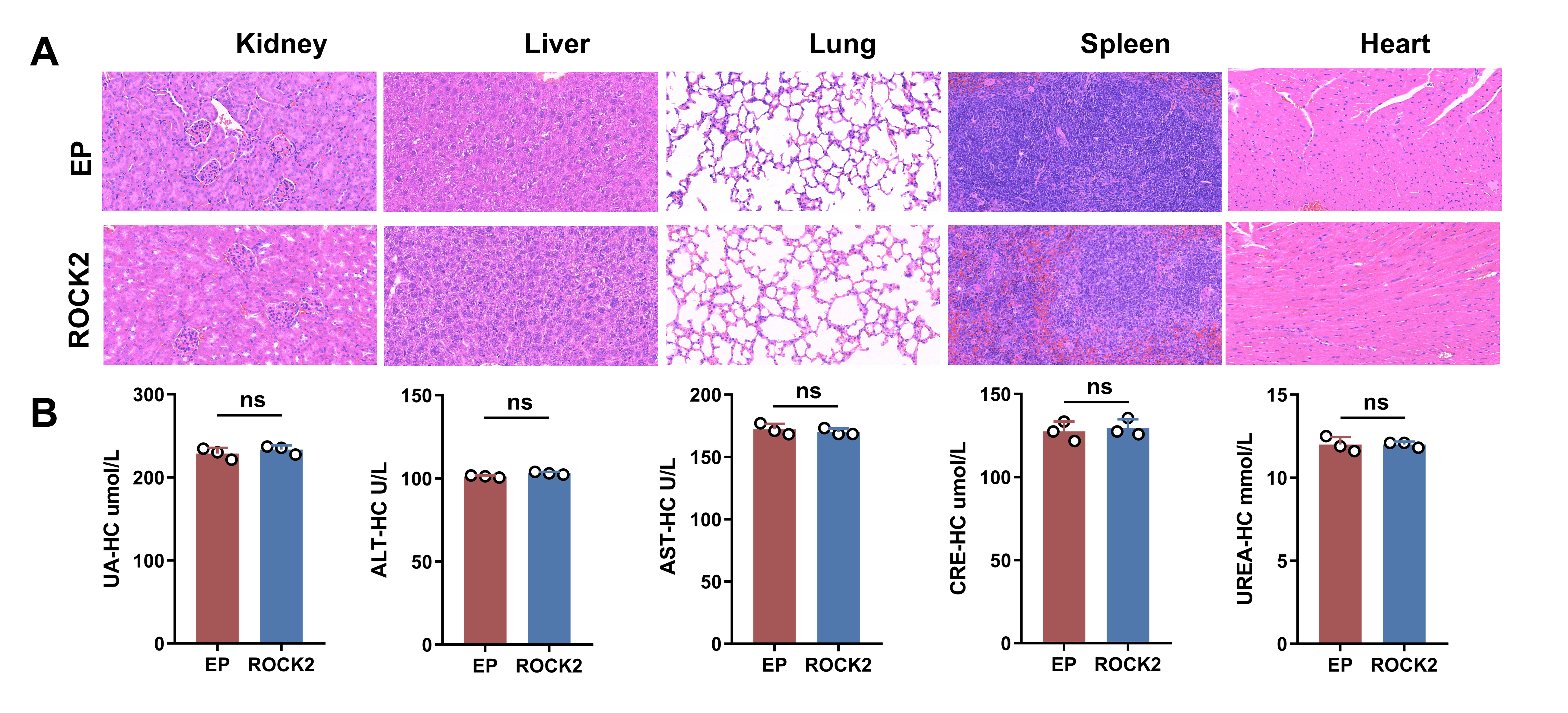
Fig. S10 Histopathological and serological assessment of belumosudil biosafety in TLE mouse models.** (A) Representative HE staining of major organs (kidney, liver, lung, spleen, and heart) from epilepsy model (EP) and belumosudil-treated (ROCK2) mice. (B) Serum biochemical profiles comparing EP and ROCK2 groups, including uric acid (UA), alanine aminotransferase (ALT), aspartate aminotransferase (AST), creatinine (CRE), and urea (UREA) levels.

**Supplementary Tables**

Table S1. Demographic and clinical information of TLE patients and controls in PET cohorts.

|  | HC group (n=23) | TLE group (n=25) | *P*-value |
| --- | --- | --- | --- |
|  |  |  |  |
| Sex (Female/Male) | 9/14 | 8/17 | 0.7640 |
| Age (years) | 30.48±14.65 | 31.16±11.30 | 0.8569 |
| Education (years) | 11.83±4.499 | 10.36±3.872 | 0.2313 |
| Result of MRI (positive/negative) | 0/23 | 20/5 | <0.0001 |
| Age at seizure onset (years) | NA | 16.04±14.58 | NA |
| Monthly seizure frequency | NA | 8.740±18.12 | NA |
| Duration (years) | NA | 14.28±10.60 | NA |
| Number of drugs | NA | 1.52±0.71 | NA |
| TLE laterality, %left | NA | 17(68%) | NA |

Note: Data are mean±SD. *P*-values are for unpaired t-tests (continuous variables) or χ2 (categorical variables).

Abbreviations: HC, healthy controls; TLE, temporal lobe epilepsy; NA, not applicable.

Table S2. Demographic and clinical information of TLE patients and autopsy controls in transcriptomics cohorts.

| Subjects | Age | Sex | rRNA proportions | RIN | Post-mortem interval |
| --- | --- | --- | --- | --- | --- |
| PD-1 | 31 | M | 1.2 | 8.7 | 7 |
| PD-2 | 60 | M | 0.6 | 6.6 | 14 |
| PD-3 | 67 | M | 1.1 | 8.4 | 6 |
| PD-4 | 77 | M | 0.5 | 5.4 | 12 |
| PD-5 | 50 | F | 1.1 | 7 | 8 |
| PD-6 | 14 | F | 1.4 | 7.9 | 10 |
| TLE-1 | 27 | M | 0.4 | 6.3 |  |
| TLE-2 | 20 | M | 0.3 | 6.6 |  |
| TLE-3 | 30 | M | 0.5 | 5.3 |  |
| TLE-4 | 42 | F | 1.4 | 7 |  |
| TLE-5 | 23 | M | 0.2 | 6.7 |  |
| TLE-6 | 26 | M | 0.4 | 7.1 |  |

Abbreviations: PD, postmortem donor; TLE, temporal lobe epilepsy; RIN, RNA integrity number.

Table S3. Significantly decreased nodal connectivity strength of the synaptic density connectome in TLE patients (top 30).

| Regions | Lobe | Gyrus | t-value | P_FDR_-value |
| --- | --- | --- | --- | --- |
| PhG_L_6_1 | Temporal lobe | Parahippocampal gyrus | -4.835738 | 0.000172 |
| ITG_L_7_5 | Temporal lobe | Inferior temporal gyrus | -4.834258 | 0.000172 |
| ITG_R_7_6 | Temporal lobe | Inferior temporal gyrus | -4.832905 | 0.000172 |
| MTG_R_4_1 | Temporal lobe | Middle temporal gyrus | -4.607072 | 0.000307 |
| MTG_R_4_4 | Temporal lobe | Middle temporal gyrus | -4.418040 | 0.000493 |
| Amyg_L_2_1 | Subcortical nuclei | Amygdala | -5.809796 | 0.000048 |
| Amyg_R_2_1 | Subcortical nuclei | Amygdala | -4.920529 | 0.000166 |
| Hipp_L_2_2 | Subcortical nuclei | Hippocampus | -5.794018 | 0.000048 |
| Tha_R_8_5 | Subcortical nuclei | Thalamus | -5.472087 | 0.000053 |
| BG_L_6_1 | Subcortical nuclei | Basal ganglia | -5.627795 | 0.000051 |
| BG_R_6_1 | Subcortical nuclei | Basal ganglia | -5.230948 | 0.000083 |
| BG_R_6_4 | Subcortical nuclei | Basal ganglia | -5.093633 | 0.000121 |
| BG_R_6_3 | Subcortical nuclei | Basal ganglia | -4.744905 | 0.000220 |
| MFG_L_7_4 | Frontal lobe | Middle frontal gyrus | -5.623267 | 0.000051 |
| MFG_R_7_3 | Frontal lobe | Middle frontal gyrus | -5.577060 | 0.000051 |
| MFG_R_7_4 | Frontal lobe | Middle frontal gyrus | -4.937688 | 0.000166 |
| MFG_R_7_6 | Frontal lobe | Middle frontal gyrus | -4.896225 | 0.000170 |
| MFG_R_7_2 | Frontal lobe | Middle frontal gyrus | -4.510166 | 0.000382 |
| IFG_R_6_2 | Frontal lobe | Inferior frontal gyrus | -4.686311 | 0.000256 |
| PrG_R_6_6 | Frontal lobe | Precentral gyrus | -5.038926 | 0.000136 |
| OrG_R_6_6 | Frontal lobe | Orbital gyrus | -4.607534 | 0.000307 |
| PCun_L_4_4 | Parietal lobe | Precuneus | -6.543734 | 0.000011 |
| PoG_R_4_2 | Parietal lobe | Postcentral gyrus | -5.444970 | 0.000053 |
| PoG_R_4_1 | Parietal lobe | Postcentral gyrus | -5.323668 | 0.000066 |
| IPL_R_6_6 | Parietal lobe | Inferior parietal lobule | -4.506461 | 0.000382 |
| LOcC_R_2_1 | Occipital lobe | Lateral occipital cortex | -5.461074 | 0.000053 |
| MVOcC_L_5_5 | Occipital lobe | MedioVentral occipital cortex | -5.376344 | 0.000061 |
| MVOcC_R_5_5 | Occipital lobe | MedioVentral occipital cortex | -5.009541 | 0.000140 |
| CG_L_7_7 | Limbic lobe | Cingulate gyrus | -4.833917 | 0.000172 |
| CG_L_7_6 | Limbic lobe | Cingulate gyrus | -4.584138 | 0.000319 |

Table S4. Statistical analysis for HADDOCK generated LC3 and ROCK2 docked complexes.

| S.No. | Cluster | HADDOCK scorea(a.u.) | Cluster Size | RMSD from overall lowest-energy structure (Å) | Vander Waals energy (Evdw) (kcal mol-1) | Electrostatic energyb(Eelec) (kcal mol-1) | Desolvation energy (Edesol) (kcal mol-1) | Restraints violation energy (kcal mol-1) | Buried surface area (Å2) | Z-Score |
| --- | --- | --- | --- | --- | --- | --- | --- | --- | --- | --- |
| 1 | 3 | 209.9 +/- 21.3 | 6 | 16.1 +/- 0.3 | -102.4+/- 11.5 | -350.0 +/- 70.4 | 8.0 +/- 2.2 | 3743.0 +/- 167.0 | 3374.7 +/- 449.9 | -1.5 |
| 2 | 2 | 230.0 +/- 15.1 | 6 | 19.3 +/- 0.2 | -105.2 +/- 5.6 | -273.2 +/- 76.1 | -9.1 +/- 8.9 | 3989.4 +/- 168.2 | 3766.1 +/- 127.8 | -0.7 |
| 3 | 1 | 257.7 +/- 36.2 | 6 | 24.0 +/- 0.1 | -76.0 +/- 7.9 | -438.3 +/- 68.2 | 27.7 +/- 4.9 | 3937.0 +/- 239.5 | 2985.9 +/- 227.6 | 0.5 |
| 4 | 4 | 258.1 +/- 2.0 | 5 | 19.4 +/- 0.1 | -101.1 +/- 6.0 | -182.7 +/- 65.3 | -17.8+/- 6.9 | 4135.6 +/- 60.4 | 3616.9 +/- 65.9 | 0.5 |
| 5 | 5 | 276.8 +/- 40.9 | 4 | 20.4 +/- 0.3 | -62.8 +/- 2.4 | -288.3 +/- 115.9 | -2.4 +/- 4.5 | 3996.3 +/- 365.3 | 3065.8 +/- 210.9 | 1.3 |

Note: The HADDOCK score = Evdw+ Eelec+ EAIR; In the equation, Evdw and Eelecrepresentvan der Waals and electrostatic energies, respectively. Whereas, EAIRindicates distance restraint contribution of AIRs. After the water refinement, the HADDOCK score was calculated as the following weighted sum: HADDOCK score = 1.0Evdw + 0.2Eelec + 1.0Edist + 0.1Esolv. Where, Esolv;solvationandEdist; distance restraints energies include both unambiguous interaction restraints and AIRs. b. Non-bonded interactions were calculated with the Optimized Potentials for Liquid Simulations (OPLS) force field using 8.5Å cut-off.
